# Activation of the Nrf2 Cell Defense Pathway by Ancient Foods: Disease Prevention by Important Molecules and Microbes Lost from the Modern Western Diet

**DOI:** 10.1371/journal.pone.0148042

**Published:** 2016-02-17

**Authors:** Donald R. Senger, Dan Li, Shou-Ching Jaminet, Shugeng Cao

**Affiliations:** 1 Department of Pathology and Center for Vascular Biology Research, Beth Israel Deaconess Medical Center, Boston, Massachusetts, United States of America; 2 Department of Pathology, Harvard Medical School, Boston, Massachusetts, United States of America; 3 Department of Pharmaceutical Sciences, Daniel K. Inouye College of Pharmacy, University of Hawaii at Hilo, Hilo, Hawaii, United States of America; North Carolina State University, UNITED STATES

## Abstract

The Nrf2 (NFE2L2) cell defense pathway protects against oxidative stress and disorders including cancer and neurodegeneration. Although activated modestly by oxidative stress alone, robust activation of the Nrf2 defense mechanism requires the additional presence of co-factors that facilitate electron exchange. Various molecules exhibit this co-factor function, including sulforaphane from cruciferous vegetables. However, natural co-factors that are potent and widely available from dietary sources have not been identified previously. The objectives of this study were to investigate support of the Nrf2 cell defense pathway by the alkyl catechols: 4-methylcatechol, 4-vinylcatechol, and 4-ethylcatechol. These small electrochemicals are naturally available from numerous sources but have not received attention. Findings reported here illustrate that these compounds are indeed potent co-factors for activation of the Nrf2 pathway both *in vitro* and *in vivo*. Each strongly supports expression of Nrf2 target genes in a variety of human cell types; and, in addition, 4-ethylcatechol is orally active in mice. Furthermore, findings reported here identify important and previously unrecognized sources of these compounds, arising from biotransformation of common plant compounds by lactobacilli that express phenolic acid decarboxylase. Thus, for example, *Lactobacillus plantarum*, *Lactobacillus brevis*, and *Lactobacillus collinoides*, which are consumed from a diet rich in traditionally fermented foods and beverages, convert common phenolic acids found in fruits and vegetables to 4-vinylcatechol and/or 4-ethylcatechol. In addition, all of the alkyl catechols are found in wood smoke that was used widely for food preservation. Thus, the potentially numerous sources of alkyl catechols in traditional foods suggest that these co-factors were common in ancient diets. However, with radical changes in food preservation, alkyl catechols have been lost from modern foods. The absence of alkyl catechols from the modern Western diet suggests serious negative consequences for Nrf2 cell defense, resulting in reduced protection against multiple chronic diseases associated with oxidative stress.

## Introduction

In all mammals, oxygen is critical for cellular metabolism and survival. However, oxygen is also a toxic gas [1]. Oxidative stress, mediated by reactive oxygen species and free radicals, chemically damages proteins, nucleic acids, lipids, and mitochondria and ultimately harms living cells, resulting in cell senescence and death [1, 2]. The serious threat of oxygen toxicity is often overlooked because all aerobic organisms, including mammals, have intrinsic mechanisms that protect against oxidative damage. Nonetheless, much evidence indicates that excessive oxidative stress directly causes or contributes to many common diseases, including cancer [3–5], coronary artery disease [3, 6], osteoporosis [7, 8], inflammatory bowel diseases [9], metabolic syndrome [3, 10], and neuro-degeneration [11–13] including Parkinson’s disease [14] and possibly also Alzheimer’s disease [3, 15, 16]. In addition, oxidative stress contributes to insulin resistance [17] and the pathological consequences of diabetes [18], kidney disease [19], multiple sclerosis [20], and aging [11, 21, 22], and can contribute to neurodevelopmental defects in the embryo [5].

In mammalian cells, the master regulator of oxidant defense is the Nrf2 (NFE2L2) pathway [23, 24]. The Nrf2 transcription factor, through binding to antioxidant response elements (AREs), induces expression of anti-oxidant and detoxifying enzymes that protect against oxidative damage and also provide protection against toxic foreign chemical substances through phase II enzyme modification [25, 26]. The network of protective genes regulated by Nrf2 is very large, with estimates of more than 1% of the total genome [27]. In the absence of oxidative stress, Nrf2 is retained in the cytosol by the actin-binding protein Keap1 that promotes rapid Nrf2 degradation by proteasomes [23, 24, 28–30]. However, under conditions of oxidative stress, Nrf2 is released from Keap1 and rapidly moves to the nucleus to induce expression of anti-oxidant and detoxifying enzymes. The “redox sensor” mechanism that releases Nrf2 from Keap1, thereby allowing Nrf2 transport to the nucleus, involves oxidation-sensitive sulfhydryl groups in cysteine residues of Keap1 [31–34].

Compelling evidence for the importance of protection provided by the Nrf2 pathway comes from numerous studies with mice lacking the Nrf2 gene. Although viable and fertile, Nrf2 null mice are considerably more sensitive to chemical carcinogens, with increased incidence of cancers demonstrated in skin, stomach, colon, bladder, and mammary gland [24, 35–39]. Nrf2 null mice also are more sensitive to a multitude of chemical toxins, resulting in increased inflammation and damage to lung, brain, and kidney [24, 40]. Furthermore, Nrf2 null mice exhibit impaired liver regeneration [41], increased susceptibility to asthma [42], reduced bone acquisition [43], defects in bone repair [44], accelerated neuro-degeneration [12, 13], accelerated UVB-induced photo-ageing of skin [45], increased rheumatoid arthritis [46], development of lupus-like autoimmune kidney disease [47], development of age-related retinopathy [48], and increased loss of skeletal muscle cells with aging [49]. Similarly to Nrf2 null mice, mice engineered with an inactive, dominant-negative Nrf2 mutant transgene develop skin cancer at three times the frequency of control mice in a classical two-stage model of chemical carcinogenesis [39]. Thus, there is considerable evidence that the Nrf2 pathway provides important protection against many common diseases, including cancer, and that Nrf2 supports the health of multiple organ systems.

**Fig 1:**
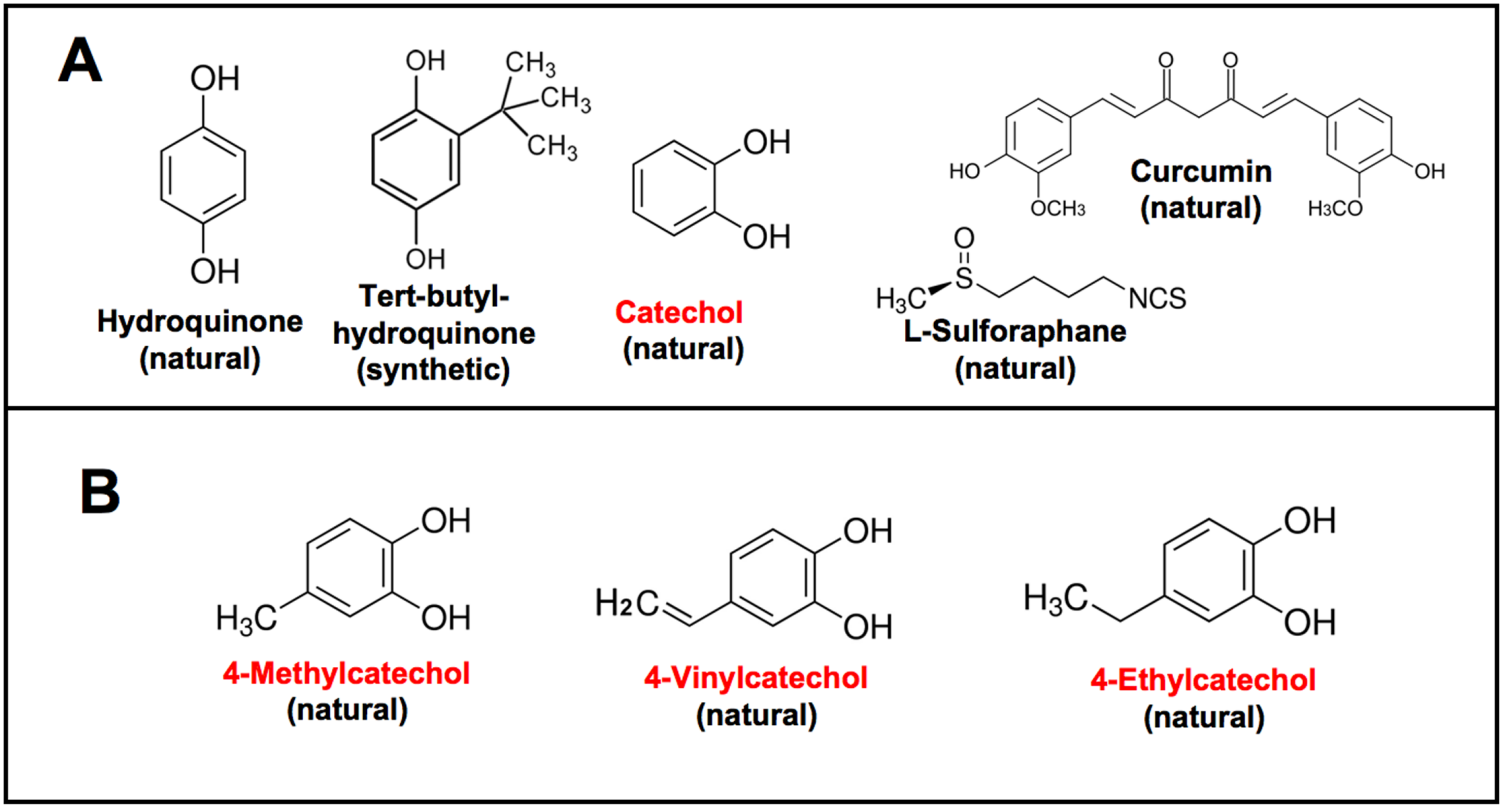
Chemical structures of Nrf2 pathway activators. **(A)** Well known synthetic and natural Nrf2 activators. **(B)** The alkyl catechol Nrf2 activators that are the focus of this study.

Although oxidative stress is sufficient for activation of the Nrf2 pathway and induction of Nrf2 target genes, activation by oxidative stress is greatly enhanced by the presence of chemical compounds with specific redox cycling properties. Originally, before the discovery of Nrf2, a variety of small chemical compounds were observed to protect rodents from chemically induced carcinogenesis [50]. Remarkably, these “cancer-protective” compounds were from distinctly different chemical classes, but they all shared the critical property of high susceptibility to oxidation-reduction reactions [51]. The simplest of these active cancer-protective compounds were identified as 1,4-benzenediol (hydroquinone), tert-butylhydroquinone, and 1,2-benzendiol (catechol), and more complex examples include the isothiocyanate sulforaphane isolated from broccoli [52, 53] and curcumin from the turmeric plant [54, 55] (see Fig 1 for structures). With the discovery of Nrf2, it became clear that these previously identified redox-cycling, cancer-protective compounds worked as important co-factors for Nrf2 activation [30, 56, 57]. Collectively, these findings suggested that support of Nrf2 activation by redox-sensitive co-factors, particularly dietary factors such as sulforaphane and curcumin, could be employed as an effective cancer prevention strategy [51–53, 56, 58–60]. This provided impetus for clinical trials [61, 62] that are continuing. Sulforaphane and curcumin are also currently in clinical trials for non-cancerous disorders in which the Nrf2 pathway has been implicated. Current challenges with the application of sulforaphane and curcumin for clinical benefits appear to involve bioavailability of these compounds [61–65].

In contrast to sulforaphane and curcumin, the naturally occurring redox-sensitive alkyl catechols, 4-methylcatechol, 4-vinylcatechol, and 4-ethylcatechol, have not received attention as potential activators of the Nrf2 pathway. Structurally similar to catechol, these alkyl catechols are unrelated to sulforaphane and curcumin (Fig 1). Also, in contrast to sulforaphane and curcumin, there are potentially numerous natural dietary sources of alkyl catechols that have been overlooked. The aim of work described here was to investigate this panel of alkyl catechols as potentially important activators of Nrf2 cell defense. Our findings illustrate that these compounds are indeed potent activators of the Nrf2 pathway both *in vitro* and *in vivo*. Moreover, our findings identify important and previously unrecognized connections between activation of the Nrf2 pathway, probiotic bacteria, fermented foods, traditional diets, and traditional methods of food preservation and suggest that alkyl catechols were common in ancient diets. However, because of extensive changes in food preservation and preparation, the alkyl catechols mostly have been lost from modern Western foods. The absence of alkyl catechols from modern Western diets suggests serious negative consequences for maintenance of Nrf2 cell defense, resulting in reduced protection against chronic diseases associated with oxidative stress.

## Materials and Methods

### Antibodies

The following antibodies (Abcam, Inc., Cambridge, MA) were used for western blotting: heme oxygenase-1 (rabbit monoclonal, clone EP1391Y), Nrf2 (rabbit monoclonal, clone EP1808Y), CD31 (rabbit monoclonal, clone EPR3094). Secondary antibody used for western blotting was polyclonal goat anti-rabbit (H+L), conjugated to horseradish peroxidase (Life Technologies, cat. # G-21234). The following antibody was used for immunohistochemical staining of Nrf2 in cells [66]: Nrf2 (H-300), sc-13032 rabbit polyclonal raised against amino acids 37–336 of human Nrf2 (Santa Cruz Biotechnology Inc.). Secondary antibody used for immunohistochemical staining was goat anti-rabbit IgG (H+L) Alexa Fluor 488 conjugate (ThermoFisher).

### Chemicals

All chemicals in pure form were purchased from Sigma-Aldrich with the exception of L-sulforaphane and luteolin from Cayman Chemical, quercetin from Tocris Bioscience, and 4-vinylcatechol from Toronto Research Chemicals. Because 4-vinylcatechol (also known as 3,4-dihydroxystyrene) contains a vinyl group (see Fig 1) it polymerizes in the presence of oxygen, similarly to other styrenes. To prevent polymerization, the supplier (Toronto Research Chemicals), provides this compound under inert atmosphere together with 1% w/w butylated hydroxytoluene. In control experiments, we determined that this proportion of butylated hydroxytoluene had no affect on results. Once a vial of 4-vinylcatechol is opened to room air and suspended in water, the integrity of the stock compound begins to deteriorate, and we found it best to open a new vial for each experiment. Dilute 4-vinylcatechol (30 μM, as used in cell culture experiments) is much more stable because the low concentration disfavors polymerization. In general, catechol and alkyl catechols were prepared as 3mM stock solutions in purified water as vehicle, filter sterilized, and diluted to the final concentration indicated for each experment.

### Cells and cell culture experiments, cell viability assays, RNA isolation from cells, immunohistochemical staining of cells, and cell lysates for western blotting

Primary human dermal microvascular endothelial cells, human umbilical vein endothelial cells, human astrocytes, and human dermal keratinocytes were purchased from Lonza Inc, Walkersville MD, and cultured in Lonza media as follows: EGM-2MV for all endothelial cells, KGM-Gold for keratinocytes, AGM for astrocytes. Keratinocytes were used for experiments at passage 3; astrocytes at passage 4, and endothelial cells at passage 6 or less. Cell viability was measured with the LIVE/DEAD Viability/Cytotoxicity Kit for mammalian cells from Molecular Probes. This method uses calcein AM (green fluorescence) to mark live cells and ethidium homodimer-1 (red fluorescence) to stain the nuclei of cells with damaged membranes. For each data point, at least four random microscopic fields containing at least 250 cells/field were photographed and enumerated.

For RNA, cells were cultured in 6 well plates, and RNA isolated with the Qiagen RNeasy Plus Mini Kit that provides on-column removal of genomic DNA. For immunohistochemical staining of Nrf2, cells were fixed and stained exactly as described [66]. In addition, cells were co-stained for F-actin with Alexa Fluor 488 phalloidin (Molecular Probes, #A12379). All images used in comparisons were captured with identical exposures. For western blotting applications, cells were cultured in 24 well plates, treated as described in figure legends, washed 3x with ice cold PBS, and harvested in 120 μL of 1.5x Laemmli SDS sample buffer containing protease inhibitor cocktail (Sigma-Aldrich, #P8340). Unless indicated otherwise, cells were cultured at 37°C in a standard tissue culture incubator in room air supplemented with 5% CO2. However, for hypoxia experiments, cells were cultured at 37°C in atmosphere consisting of 5% CO2, 2% oxygen, and 93% nitrogen.

### Quantitative real-time PCR (RT-PCR)

Multi-gene transcriptional profiling with quantitative RT-PCR was performed with SYBR Green I and the Applied Biosystems 7500 Fast Real-Time PCR System, as described [67]. cDNA was prepared with SuperScript III reverse transcriptase (Life Technologies) [67]. For primer design and validation, gene-specific sequences were selected based on NCBI Nucleotide BLAST searches to eliminate any homology to other genes [68]. In addition, primers satisfied standard design parameters of Primer Express Software (Applied Biosystems). Primers were synthesized by Integrated DNA Technologies (Coraville, IA); for primer sequences see S1 Table. All PCR reactions were performed in duplicate and copy numbers were calculated from standard curves generated from a master template [68]. Mean and standard deviation (S.D) were calculated from at least three different experimental samples. Data were expressed as copy number/10^6^ copies of 18S rRNA that is superior to using housekeeping genes for normalizing across multiple cell types [67, 69, 70]. Also, mRNAs encoding control housekeeping genes, appropriate to each cell type, were also measured; and data are presented in each of the figures. Thus, two levels of normalization control are provided in each of the figures presenting RT-PCR data: (i) mRNA copy number for gene of interest/10^6^ copies 18S rRNA, and (ii) direct comparisons between mRNA copy number for genes of interest vs. relevant housekeeping genes, each expressed as a function of mRNA copy number/10^6^ copies 18S rRNA.

### Statistical analyses and assignment of statistical significance

All data are presented as mean ± S.D. Statistical analyses were performed with GraphPad software using the unpaired t-test. In all cases, an individual experimental group was compared with the appropriate vehicle control group; and calculated p-values are based on direct comparisons between the two groups. For assignment of statistical significance based on p values, standard threshold limits were used: p < 0.001 extremely significant, p = 0.001 to < 0.01 very significant, p = 0.01 to < 0.05 significant, p ≥ 0.05 not significant.

### Western blotting

Cell lysates subjected to SDS polyacrylamide electrophoresis on pre-cast 4–20% gradient gels (GenScript) and separated proteins transferred to PVDF immunoblotting membrane (Bio-Rad). PVDF membrane was blocked in 5% w/v skim milk (from powder, EMD Millipore), and stained with primary antibodies and secondary horseradish peroxidase-conjugated antibody (see Antibodies, above). Signal was developed with ECL western blotting substrate (Pierce Chemical) and captured on x-ray film. For re-probing with additional antibodies, blots were stripped (Re-Blot Plus, Millipore) and blocked again with skim milk, as above.

### siRNA experiments

Selective inhibition of Nrf2 (NFE2L2) expression with small interfering RNAs (siRNAs) was accomplished with a predesigned human NFE2L2 TriFECTa RNAi Kit containing Dicer-substrate 27mer siRNA duplexes from Integrated DNA Technologies (cat. ID: HSC.RNAI.N001145413.12). For these experiments we used human umbilical vein endothelial cells (see above) because they are particularly well suited for transfection that was performed with the Lipofectamine RNAiMAX Reagent in Opti-MEM Reduced Serum Medium (both from Life Technologies) according to manufacturer’s instructions. Two Nrf2 (NFE2L2) siRNA duplexes were highly effective: NM_001145423 duplex 1 and NM_001145413 duplex 2, each at a final concentration of 1 nM. Another duplex, DS NC1 (provided with the kit) served as negative control.

### Mice, mouse diet, and RNA isolation from mouse tissues

This study was carried out in strict accordance with the recommendations in the Guide for the Care and Use of Laboratory Animals of the National Institutes of Health. The Beth Israel Deaconess Medical Center Institutional Animal Care and Use Committee (IACUC) specifically approved this study (protocol #045–2012). All efforts were made to minimize suffering; euthanasia was performed according to National Institutes of Health guidelines.

Retired breeder male BALB/c mice (Charles River Laboratories, age ~ 7 months) were used for these experiments. Mice were placed on the purified rodent diet AIN 76A (Research Diets, Inc.) for 3–4 weeks prior to administration of alkyl catechols, as described in figure legends. Kidney and lung were minced and submerged in RNAlater and RNA isolation performed with a Polytron tissue homogenizer and the RNeasy Lipid Tissue Mini Kit (Qiagen) that employs on-column removal of DNA with DNAse digestion (RNase-free DNAse set from Qiagen).

### Lactobacilli and biotransformation of chlorogenic acid, caffeic acid, 3,4-dihydroxybenzoic acid; and chromatographic analyses

All lactobacillus strains were purchased from the American Type Culture Collection (ATCC), Rockville MD. Strain designations are as follows: *L*. *plantarum* (ATCC 8014), *L*. *brevis* (ATCC 8287), *L*. *collinoides* (ATCC 27611), *L*. *reuteri* MM4-1A (ATCC PTA-6475), *L*. *ruminus* (ATCC 27780), and *L*. *paracaseii* (ATCC 25302). All were grown in Lactobacilli MRS broth (Difco) according to the specific instructions provided.

Solutions of chlorogenic acid, caffeic acid, and 3,4-dihydroxybenzoic acid were prepared to a final concentration of 6 mM by dissolving in PBS containing 10 mg/ml D-glucose (PBS-glucose). Each was filter sterilized with Steriflip 0.22 micron filter units (EMD-Millipore). Lactobacilli were concentrated by centrifugation, washed twice with sterile PBS-glucose, and incubated at a final density of ~ 8 x 10^8^ bacterial cells/ml with the various solutions, including PBS-glucose control, on a rocker for 24 hours at room temperature. Supernatants were harvested by centrifugation and filter-sterilized before adding to endothelial cells for Nrf2 assays with RT-PCR and western blotting.

Reversed-phase high-pressure liquid chromatography (HPLC) was performed with a Phenomenex Luna 5 micron C18 column (100x4.6mm) using a 20 minute gradient consisting of 10%-100% acetonitrile (in 0.1% formic acid). Standards were dissolved in methanol (1 mg/mL) and volume injected was 10 μL.

## Results

### Activation of the Nrf2 pathway by alkyl catechols *in vitro* and *in vivo*

#### Nrf2 pathway activation, as measured by induction of Nrf2 target genes in human endothelial cells, astrocytes, and keratinocytes

**Fig 2:**
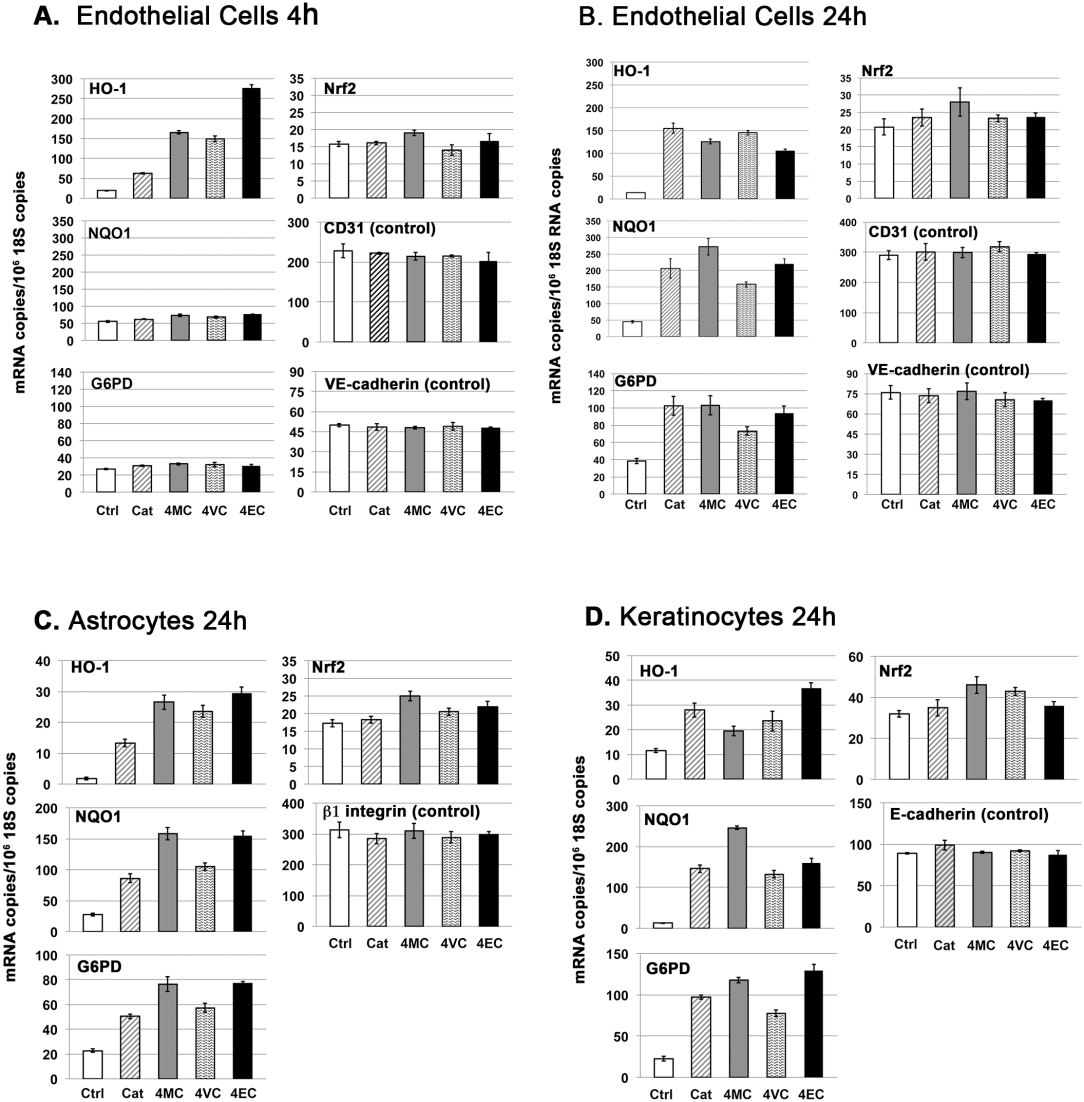
Induction of Nrf2 target gene mRNAs by alkyl catechols and catechol, as measured with RT-PCR. Y-axis = (mRNA copies)/(10^6^ 18S rRNA copies). Nrf2 target genes = heme oxygenase-1 (HO-1), NAD(P)H:quinone oxidoreductase 1 (NQO1), glucose 6-phosphate dehydrogenase (G6PD). Control non-NRF2 target mRNAs relevant to each cell type also were measured: CD31 (PECAM-1), VE-cadherin (cadherin-5), integrin subunit β1, and E-cadherin (cadherin-1). Test compounds were added to a final concentration of 30 μM and cells harvested at the time indicated. Ctrl = vehicle (H_2_O) control, Cat = catechol, 4MC = 4-methylcatechol, 4VC = 4-vinylcatechol, 4EC = 4-ethylcatechol. **(A)** Human dermal microvascular endothelial cells at 4 hours; **(B)** Human dermal microvascular endothelial cells at 24 hours; **(C)** Human brain astrocytes at 24 hours; **(D)** Human dermal keratinocytes at 24 hours. For all panels, error bars = ± standard deviation (S.D.); n ≥ 3 for each data point. ***Summary of data analyses and statistical significance (see*** Methods): It should be emphasized here that the Nrf2 pathway is activated primarily by stabilization of Nrf2 protein that allows for transcriptional induction of Nrf2 target genes, such as HO-1, NQO1, and G6PD; and therefore, these target gene mRNAs are indicators of Nrf2 pathway activation. Activation of the Nrf2 pathway is not mediated primarily by induction of Nrf2 mRNA, but Nrf2 mRNA induction may contribute modestly as suggested by data shown here (see text for further explanation and references). **(Panel A)** For HO-1, individual comparisons between vehicle Ctrl and each of the other experimental conditions = all extremely significant (p<0.0001); for NQO1, G6PD, Nrf2, CD31, and VE-cadherin data sets, differences between vehicle Ctrl and each of the other experimental conditions = all not statistically significant. **(Panels B, C, D)** For HO-1, NQO1, and G6PD, differences between vehicle Ctrl and each of the other conditions = all extremely significant (p<0.0004 to p<0.0001). In contrast, for non-Nrf2 target gene controls (CD31, VE-cadherin, β1 integrin, and E-cadherin), differences between vehicle Ctrl and each of the other experimental conditions = not statistically significant. For Nrf2 in Panel B, small but statistically significant differences were observed between vehicle Ctrl and 4MC (p < 0.026) and vehicle Ctrl and 4EC (p < 0.047); for Nrf2 in Panel C, small but significant differences were observed between vehicle Ctrl and 4MC (p < 0.01) and vehicle Ctrl and 4EC (p < 0.01); for Nrf2 in Panel D, small but significant differences were observed between vehicle Ctrl and 4MC (p < 0.01) and 4VC (p < 0.02).

Nrf2 is a transcription factor, and as summarized in the Introduction, activation of the Nrf2 pathway requires: (i) stabilization of Nrf2 protein, (ii) transport of Nrf2 protein to the cell nucleus, and (iii) transcriptional activation of Nrf2 target genes. Consequently, measure of Nrf2 target gene expression is required for determination of Nrf2 pathway activation, and analyses of nuclear concentration of Nrf2 can provide complementary information. Therefore, as described here, we employed multi-faceted strategy for analyzing Nrf2 pathway activation by alkyl catechols, including: (i) real-time PCR (RT-PCR) of mRNAs encoding three prominent Nrf2 target genes: heme oxygenase-1 (HO-1), NAD(P)H:quinone oxidoreductase1 (NQO1), and glucose-6-phosphate dehydrogenase (G6PD), (ii) western blotting for protein expression of the prominent Nrf2 target gene HO-1, (iii) immunohistochemical analyses of Nrf2 protein to visualize nuclear localization, and (iv) transfection with Nrf2 siRNA to demonstrate Nrf2-dependence of target gene induction. The three Nrf2 target genes chosen for investigation here, HO-1, NQO1, and G6PD, each serve critically important functions. Mice lacking HO-1 are more susceptible to myocardial infarction [71] and atherosclerosis [72]; mice lacking NQO1 are more susceptible to chemically induced carcinogenesis [73] and chemical toxicity [74], and mice lacking G6PD exhibit increased renal oxidative stress [75] and are more susceptible to myocardial dysfunction [76]. We began by investigating induction of Nrf2 target genes by alkyl catechols in primary cultures of human microvascular endothelial cells, brain astrocytes, and dermal keratinocytes, and obtained similar results with all cell types. As shown in Fig 2, the alkyl catechols and catechol, each at a final concentration of 30 μM, markedly and selectively induced expression of HO-1, NQO1, and G6PD mRNAs in human endothelial cells, astrocytes, and keratinocytes; and statistical analyses indicated that these inductions were extremely significant. Induction of HO-1 mRNA in endothelial cells occurred by 4 hours (Fig 2 panel A), before detectable induction of NQO1 and G6PD. By 24 hours, HO-1, NQO1, and G6PD mRNAs were induced multi-fold in all cell types with extreme statistical significance (Fig 2, panels B, C, D). For each experiment, expression of the Nrf2 target gene mRNAs (HO-1, NQO1, and G6PD) were normalized to 18S rRNA copy number, and the strong inductions observed were validated additionally by measuring control non-Nrf2 target gene mRNAs encoding structural proteins, e.g. CD31 and VE-cadherin for endothelial cells (Fig 2A and 2B), β1 integrin for astrocytes (Fig 2C), and E-cadherin for keratinocytes (Fig 2D). Thus, marked induction of Nrf2 target gene mRNAs (HO-1, NQO1, G6PD), as shown in the bar graphs, was internally controlled in each sample by normalization to 18S rRNA and secondarily by measuring one or more control non-Nrf2 target gene mRNAs. Although copy numbers of the control non-Nrf2 target gene mRNAs (CD31, VE-cadherin, β1 integrin, E-cadherin) sometimes varied slightly from sample to sample (always < 10% and not statistically significant), normalization of the data to these non-Nrf2 target genes instead of 18S rRNA would have little or no impact on the magnitude of Nrf2 target gene mRNA induction (*i*.*e*., HO-1, NQO1, G6PD) depicted in the Fig 2 bar graphs.

In contrast to Nrf2 target genes HO-1, NQO1, and G6PD, we observed a very modest but still statistically significant induction of Nrf2 mRNA by 4-methylcatechol and, in some cases, by 4-vinylcatechol and 4-ethylcatechol (Fig 2). This relatively modest induction of Nrf2 mRNA (~5 to 45%, depending on alkyl catechol and cell type) likely reflects the presence of anti-oxidant response element-like sequence in the Nrf2 gene promoter that enables stabilized Nrf2 protein to induce expression of its own mRNA [77]. Regardless, it should be emphasized that activation of the Nrf2 pathway is not mediated primarily by induction of Nrf2 mRNA; rather, Nrf2 pathway activation requires Nrf2 protein stabilization, Nrf2 protein transport to the nucleus, and Nrf2 protein binding to anti-oxidant response element sequences in Nrf2 target genes (reviewed in the Introduction). Consistent with this mechanism of Nrf2 pathway activation, the Nrf2 target genes HO-1, NQO1, and G6PD each were induced by alkyl catechols with magnitudes far greater than that expected from any observed increases in Nrf2 mRNA (Fig 2A–2D). Nonetheless, the data suggest that induction of Nrf2 mRNA, particularly by 4-methylcatechol, may contribute to induction of mRNAs encoding the Nrf2 target genes HO-1, NQO1, and G6PD. Finally, and consistent with lack of cell toxicity, neither catechol nor the akyl catechols at the 30 μM concentration employed in these experiments had any adverse effect on cell viability (see S1 Fig). Furthermore, and consistent with activation of the Nrf2 pathway that protects against oxidative stress, we found that catechol and the akyl catechols each strongly improved cell survival in the presence of hydrogen peroxide (see S2 Fig).

**Fig 3:**
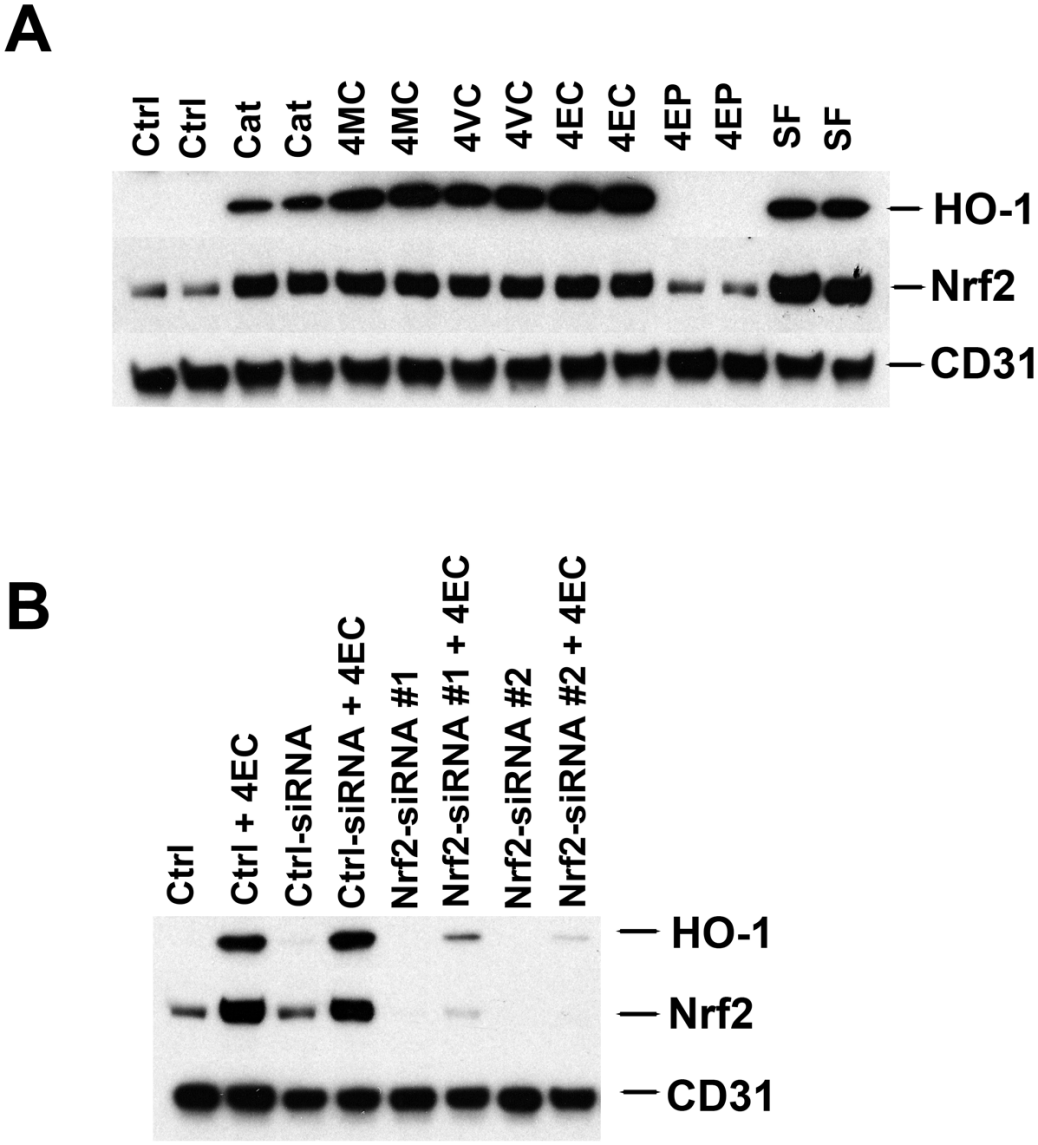
Induction of HO-1 protein expression by catechol, akyl catechols, and sulforaphane; inhibition by Nrf2 siRNAs. **(A)** Western blotting of human microvascular endothelial cells, harvested 24 hours after adding compounds. Ctrl = control, Cat = catechol, 4MC = 4-methylcatechol, 4VC = 4-vinylcatechol, 4EC = 4- ethylcatechol, 4EP = 4-ethylphenol, SF = sulforaphane. Catechol, 4MC, 4VC, 4EC, and 4EP were each added to a final concentration of 30 μM; sulforaphane was added to a final concentration of 20 μM that is the maximum tolerated dose (higher doses cause cell death). CD31 = protein loading control. (**B)** Western blotting of human umbilical vein endothelial cells, either untransfected (Ctrl) or transfected with control siRNA (ctrl-siRNA) or Nrf2 siRNAs (Nrf2-siRNA#1; Nrf2-siRNA#2)) and harvested 24 hours after addition of 30 μM 4EC (+4EC), where indicated. CD31 = protein loading control.

Next, with western blotting of human endothelial cells, we examined induction of HO-1 protein expression by each of the alkyl catechols, catechol, sulforaphane (positive control), and 4-ethylphenol (negative control). Cells were harvested 24 hours after addition of each of the compounds to 30 μM final concentration, except 20 μM for sulforaphane (SF) that we found is the maximum tolerated dose of this compound for endothelial cells (see S1 Fig). As shown in Fig 3A, all compounds except the negative control, 4-ethylphenol (4EP), strongly induced HO-1 protein. Also, the alkyl catechols were consistently more potent than catechol for inducing HO-1 protein. This finding correlates with HO-1 mRNA expression at 4 hours (see Fig 2A) but not at 24 hours (Fig 2B) possibly because induction of HO-1 mRNA is maximum before 24 hours. Regardless, western blotting clearly indicates that alkyl catechols are potent inducers of the Nrf2 target gene HO-1 and that they are comparably potent to sulforaphane.

In addition to HO-1 protein, the alkyl catechols, catechol, and sulforaphane also consistently increased Nrf2 protein > 2.5-fold (Fig 3A, quantified with Image J). This is most likely a consequence of increased Nrf2 stabilization due to release of Nrf2 from Keap1 and reduced proteosomal degradation [23, 24, 28–30] rather than an increase in Nrf2 mRNA (compare Nrf2 protein in Fig 3A with Nrf2 mRNA copy numbers in Fig 2). Finally, we investigated the dependence of HO-1 induction on Nrf2 with specific siRNAs. As shown in Fig 3B, two different Nrf2 siRNAs were used successfully to selectively target Nrf2 expression. In both cases, 4-ethylcatechol induction of HO-1 was blocked, consistent with induction of HO-1 by the Nrf2 pathway.

#### Alkyl catechols and catechol induce nuclear concentration of Nrf2; and the specificity of alkyl catechol structure in comparison with similar but inactive natural compounds

**Fig 4:**
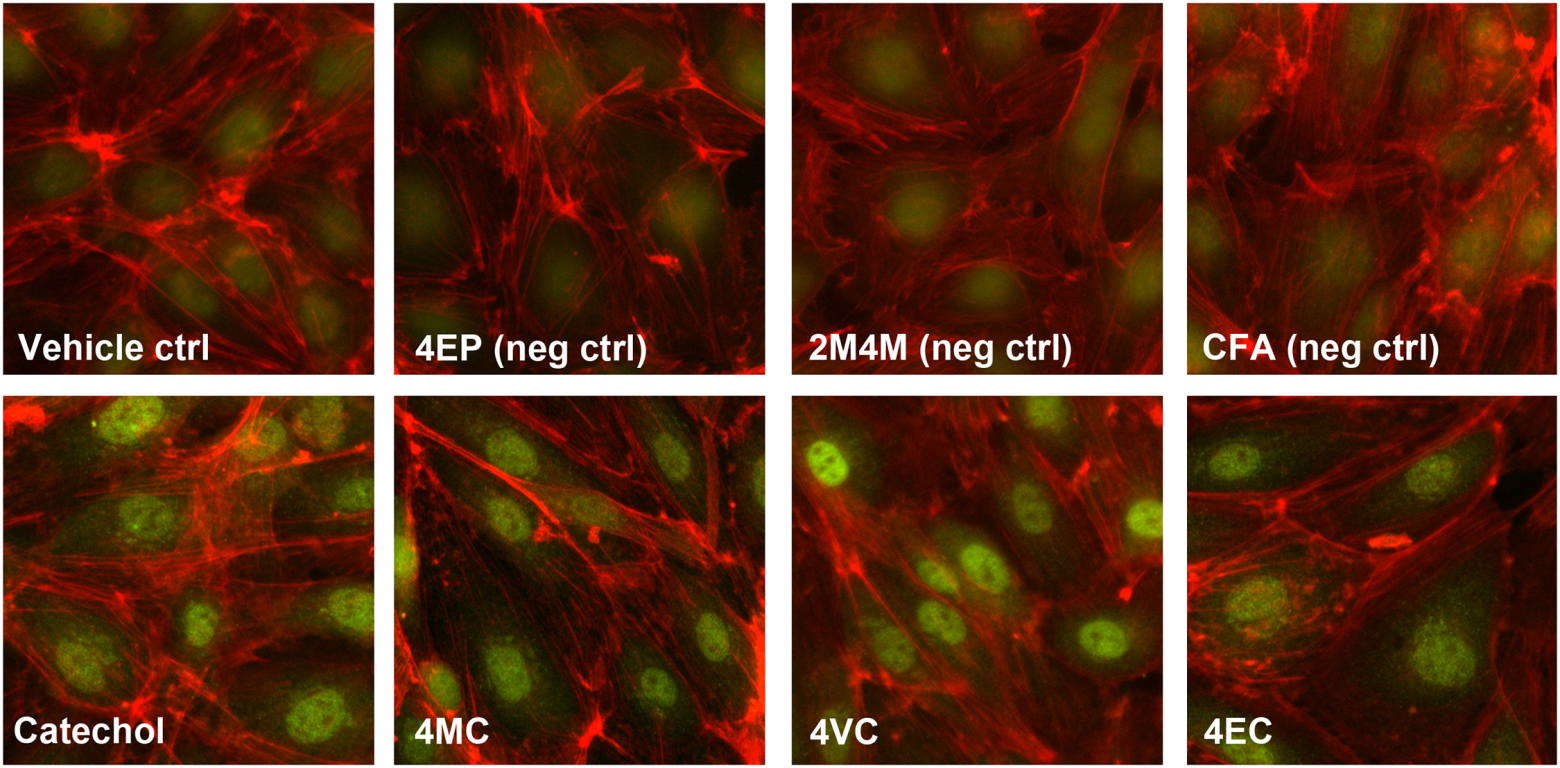
Immunohistochemical staining of Nrf2 in human endothelial cells. Cells were incubated with compounds (30 μM final concentration) for 24 hours, fixed, and stained for Nrf2 (green color) and F-actin (red color). Vehicle ctrl = vehicle control, 4EP = 4-ethylphenol, (negative control), 2M4M = 2-methoxy-4-methylphenol (negative control), CFA = caffeic acid (negative control), 4MC = 4-methylcatechol, 4VC = 4-vinylcatechol, 4EC = 4-ethylcatechol. Note bright green staining of Nrf2 in cells stimulated with catechol, 4MC, 4VC, and 4EC, in comparison with controls. See Figs 1 and 5 for all chemical structures. All samples were processed and stained in parallel; green images (Nrf2) were captured at identical exposure; and, similarly, red images (F-actin) were captured at identical exposure. Subsequently, red and green images were merged without any manipulation so that images presented here are valid for direct comparisons.

**Fig 5:**
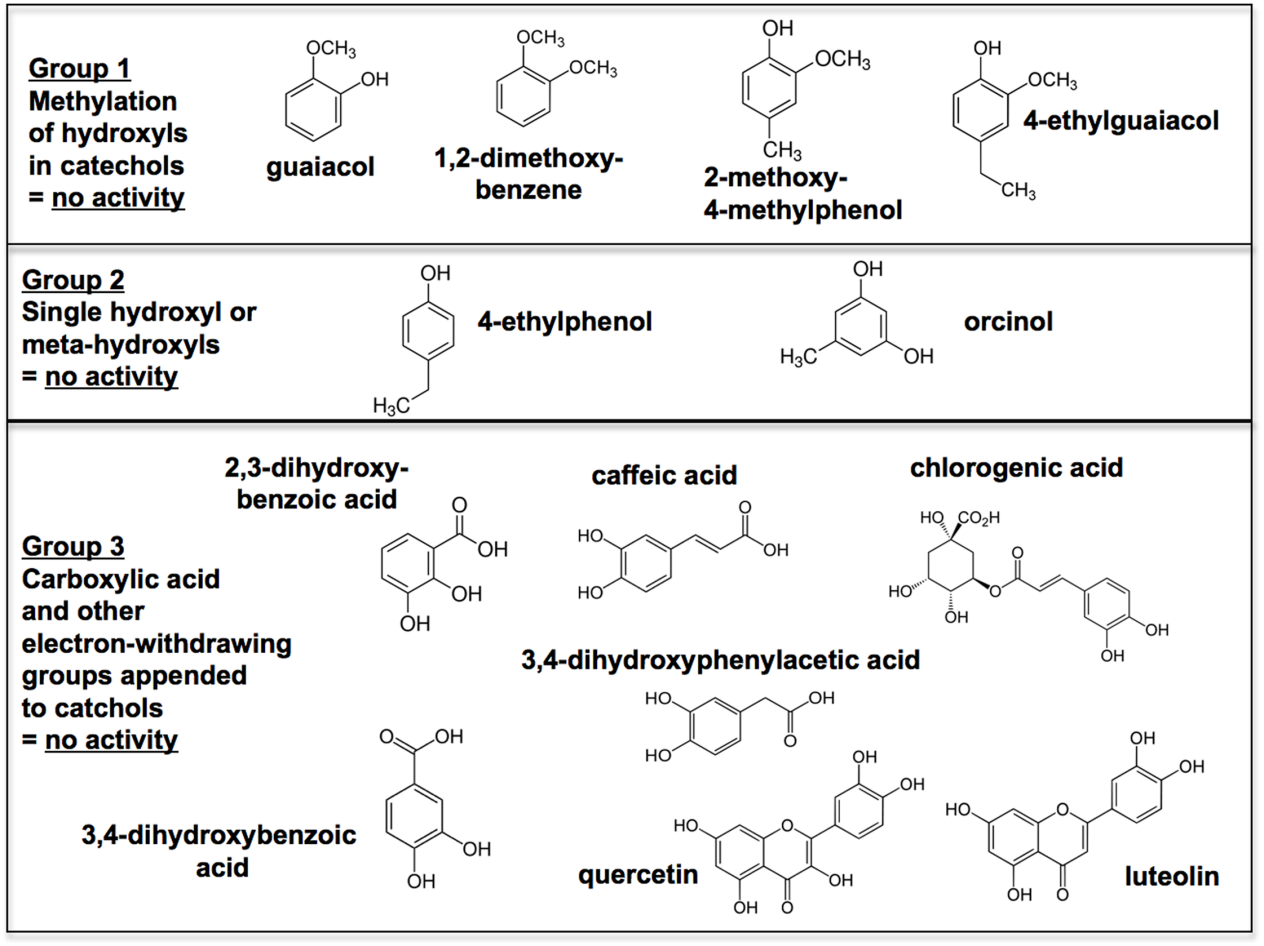
Compounds with structural similarity to catechols that do not activate the Nrf2 pathway significantly, in comparison with catechol or akyl catechols. All compounds depicted here were tested in RT-PCR assays and/or western blotting assays with human endothelial cells, as demonstrated in Figs 2 and 3 at a final concentration of 30 μM, with the exception of quercetin and luteolin that were tested at 20 μM (the maximum tolerated dose). None of these compounds induced Nrf2 target gene expression significantly in comparison with catechol or the akyl catechols. Consistent with these negative findings, each of these compounds has structural characteristics consistent with inactivity, either due to methylation of hydroxyls (top panel), lack of appropriate hydroxyls on benzene ring (middle panel), or electron-withdrawing or bulky side groups appended to the catechol moiety (bottom panel). For supporting data, see Fig 3A (for 4-ethylphenol) and subsequent figures (for caffeic acid, chlorogenic acid, and 3,4-dihydroxybenzoic acid) and also S3 Fig (for all other compounds).

Additional support for a direct link between alkyl catechols and activation of the Nrf2 pathway comes from immunohistochemical staining for Nrf2. We observed marked increases in nuclear localization of Nrf2 24 hours after addition of alkyl catechols and catechol (Fig 4 bottom panels), in comparison with negative controls (Fig 4 top panels). This increased staining of Nrf2 in the nucleus is consistent with increased expression of Nrf2 target genes HO-1, NQO1, and G6PD by alkyl catechols and catechol (Figs 2 and 3A). Increased nuclear staining for Nrf2 is also consistent with the increases in Nrf2 protein, shown in Fig 3A. The chemical structures of the inactive negative control (neg ctrl) compounds used in Fig 4, *i*.*e*., 4-ethylphenol (4EP), 2-methoxy-4-methylphenol (2M4M), and caffeic acid (CFA), are shown in Fig 5 together with other compounds that we also identified as inactive in our assays. Thus, all of the compounds depicted in Fig 5 tested negative for induction of Nrf2 target genes either with RT-PCR for HO-1, NQO1, and G6PD and/or western blotting for HO-1. As shown in Fig 3A, 4-ethylphenol does not induce HO-1 protein expression; data from several other experiments with inactive compounds are shown as negative controls in subsequent figures below and in S3 Fig. Importantly, the structures of the inactive compounds depicted in Fig 5 underscore the specificity of alkyl catechols and catechol for robust activation of the Nrf2 pathway. We found that the compounds of Group 1 (Fig 5, top panel), with methylation of one or both hydroxyl groups of the catechol moiety, lacked detectable activity, as reported previously for methylated hydroquinones [51]. Also, consistent with previous reports [51, 78], two hydroxyl groups were required for activity in our assays (e.g. 4-ethylphenol is inactive) but compounds with hydroxyls in the *meta* position (e.g. orcinol) are also inactive (Group 2). Finally, a more diverse group of compounds, containing individual catechol moieties appended either to electron-withdrawing or bulky side groups, were inactive in our assays (Group 3). Thus, collectively, our findings underscore the special importance of alkyl catechols and catechol, in comparison with a variety of related compounds.

Notably, we did not find evidence with our assays that the flavonoids quercetin or luteolin activated the Nrf2 pathway (Fig 5, S3 Fig). There are several previous reports that luteolin does not activate but rather inhibits the Nrf2 pathway [79–81]. Regarding quercetin, two previous reports claim that quercetin activates Nrf2 [82, 83] whereas another indicates that quercetin is inactive or very weak relative to classical Nrf2 inducers [84]. Explanation for the previously published, disparate claims about quercetin and the disparity between previous reports that quercetin activates Nrf2 and our negative findings (S3 Fig) might be explained by the use of different cell types and/or the use “reporter” cell lines instead of the normal cell cultures used here. Further work, beyond the scope of this project, will be required to reconcile the differences.

#### The alkyl catechols are similarly potent to sulforaphane at inducing Nrf2 target gene expression

**Fig 6:**
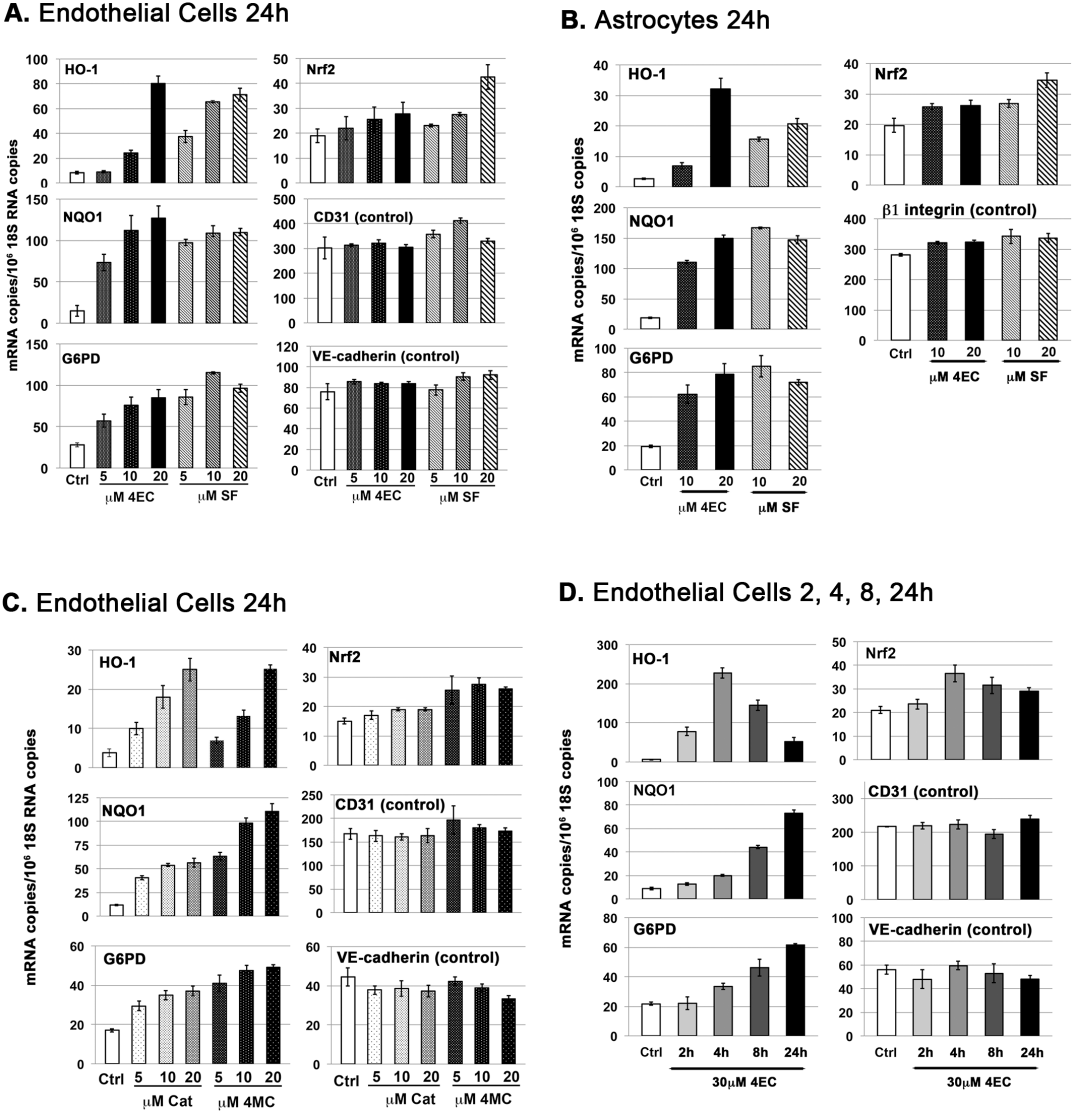
Dose comparisons of catechols and sulforaphane for induction of Nrf2 target gene expression. RT-PCR analyses; Y-axis = (mRNA copies)/(10^6^ 18S rRNA copies). Error bars = ± S.D.; n ≥ 3 for each data point. **(A)** Human dermal microvascular endothelial cells, 24 hours after stimulation with either 5, 10, 20 μM 4-ethylcatechol (4EC) or 5, 10, 20 μM sulforaphane (SF). **(B)** Human brain astrocytes stimulated for 24 hours with 4EC or SF, as in Panel A. **(C)** Human dermal microvascular endothelial cells, 24 hours after stimulation with either 5, 10, 20 μM catechol (Cat) or 5, 10, 20 μM 4-methylcatechol (4MC). **(D)** Human dermal microvascular endothelial cells stimulated with 30 μM 4EC for 2, 4, 8, or 24 hours. Nrf2 target genes = heme oxygenase-1 (HO-1), NAD(P)H:quinone oxidoreductase 1 (NQO1), glucose 6-phosphate dehydrogenase (G6PD). Control mRNAs relevant to each cell type also were measured: CD31 (PECAM-1), VE-cadherin (cadherin-5), integrin subunit β1. ***Summary of data analyses and statistical significance*:** As for Fig 2, it is important to emphasize that the Nrf2 pathway is activated primarily by stabilization of Nrf2 protein that allows for transcriptional induction of Nrf2 target genes, such as HO-1, NQO1, and G6PD; and therefore, these target gene mRNAs are indicators of Nrf2 pathway activation. Activation of the Nrf2 pathway is not mediated primarily by induction of Nrf2 mRNA, but Nrf2 mRNA induction may contribute modestly as suggested by data shown here (see text). **(Panel A)** For HO-1, individual comparisons between Ctrl and each of the other conditions indicated differences that are all extremely significant (p<0.0002), with the exception of 5 μM 4EC (not significant). For NQO1, Ctrl versus (vs.) each of the other conditions = all extremely significant (p<0.0001). For G6PD, Ctrl vs. 5 μM 4EC = very significant (p<0.002) and Ctrl vs. each of the other conditions = all extremely significant (p<0.0002). For Nrf2, modest but significant differences were observed for Ctrl vs. 20 μM 4EC (significant, p<0.05), and Ctrl vs. 20 μM SF = very significant (p<0.002); however, Ctrl vs. each of the other conditions = all not significant. For CD31 and VE-cadherin control mRNAs, Ctrl vs. each of the other conditions = all not significant. **(Panel B)** For HO-1, NQO1, and G6PD, Ctrl vs. the each of other conditions = all extremely significant (p<0.0001) with the exception Ctrl vs. 10 μM 4EC (HO-1 data) = very significant (p<0.01). For Nrf2, Ctrl vs. 10 μM, 20 μM 4EC, and 10 μM SF = all significant (p<0.03); and Ctrl vs. 20 μM SF = extremely significant (p<0.001). For β1 integrin, Ctrl vs. 10 μM 4EC and Ctrl vs. 20 μM 4EC = not significant, and Ctrl vs. 10 μM SF and Ctrl vs. 20 μM SF = significant (p<0.05). **(Panel C)** For HO-1, NQO1, and G6PD, individual comparisons for Ctrl vs. the other conditions indicated differences that are all extremely significant (p<0.0005 to p<0.0001), with the exception of Ctrl vs. 5 μM 4MC (HO1 data) = very significant (p<0.01). For Nrf2, Ctrl vs. 5 μM Cat and Ctrl vs. 5 μM 4MC = not significant; Ctrl vs. 10 μM Cat, Ctrl vs. 20 μM Cat, Ctrl vs. 10 μM 4MC = all significant (p<0.05); Ctrl vs. 20 μM 4MC = extremely significant (p<0.001). For CD31 and VE-cadherin, Ctrl vs. other experimental conditions = all not significant, with the exception of Ctrl vs. 20 μM 4MC (VE-cadherin data) = significant (p<0.05). **(Panel D)** For HO-1, NQO1, and G6PD, individual comparisons for Ctrl vs. the other conditions = all extremely significant (p<0.0001), with the exception of Ctrl vs. 2h and Ctrl vs. 4h (NQO1 data) = very significant (p<0.01), Ctrl vs. 4h and Ctrl vs. 8h (G6PD data) = significant, and Ctrl vs. 2h (G6PD data) = not significant. For Nrf2, Ctrl vs. 2h = not significant; Ctrl vs. 4h, 8h, and 24h = all significant (p<0.05). For CD31 and VE-cadherin, Ctrl vs. other experimental conditions = all not significant.

Next, we performed a series of experiments to compare the potency of alkyl catechols and catechol with sulforaphane, which is a well established and much studied activator of the Nrf2 pathway [56, 58–62, 85]. As shown in Fig 6, in direct comparison experiments, 4-ethylcatechol and sulforaphane were similarly potent at inducing Nrf2 target genes HO-1, NQO1, and G6PD in human endothelial cells (Fig 6A) and also in human astrocytes (Fig 6B). In some comparisons 4-ethylcatechol was demonstrably more potent than sulforaphane (e.g. induction of HO-1 and NQO1 in endothelial cells, Fig 6A), but in other cases sulforaphane was more potent (e.g. induction of G6PD in endothelial cells, Fig 6A). Nonetheless, on balance, the data indicate that these two compounds were similarly potent both in human endothelial cells and astrocytes. Also, both were demonstrably active at concentrations as low as 5 μM. In addition to 4-ethylcatechol and sulforaphane, both 4-methylcatechol and catechol were demonstrably active at concentrations as low as 5 μM, and each demonstrated increased activity with increased concentration (Fig 6C). We did not investigate 4-vinylcatechol in these experiments; however, as illustrated in Figs 2 and 3, 4-vinylcatechol is comparably potent to other alkyl catechols at 30 μM concentration. Finally, to investigate further the time course of induction of Nrf2 target genes HO-1, NQO1, and G6PD, 4-ethylcatechol was added to endothelial cells for 2, 4, 8, and 24 hours. RT-PCR measurement of mRNAs demonstrated marked differences in the kinetics of induction of HO-1, in comparison with NQO1 and G6PD (Fig 6D). HO-1 mRNA was maximally induced at 4 hours (38-fold), followed by a decline that, nonetheless, was still well above baseline at 24 hours (> 8-fold). In contrast, inductions of NQO1 and G6PD mRNAs were modest at 4 hours, increased by 8 hours, and increased further by 24 hours. Thus, these data illustrate distinct differences in the time course of induction of various Nrf2 target gene mRNAs and indicate gene-specific regulation in addition to Nrf2-mediated induction. They also indicate that HO-1 mRNA copy number, 24 hours following addition of 4-ethylcatechol to endothelial cells, is an underestimate of maximal induction that occurs ~ 4 hours.

#### Induction of Nrf2 target genes by alkyl catechols and catechol is co-regulated by oxygen concentration

**Fig 7:**
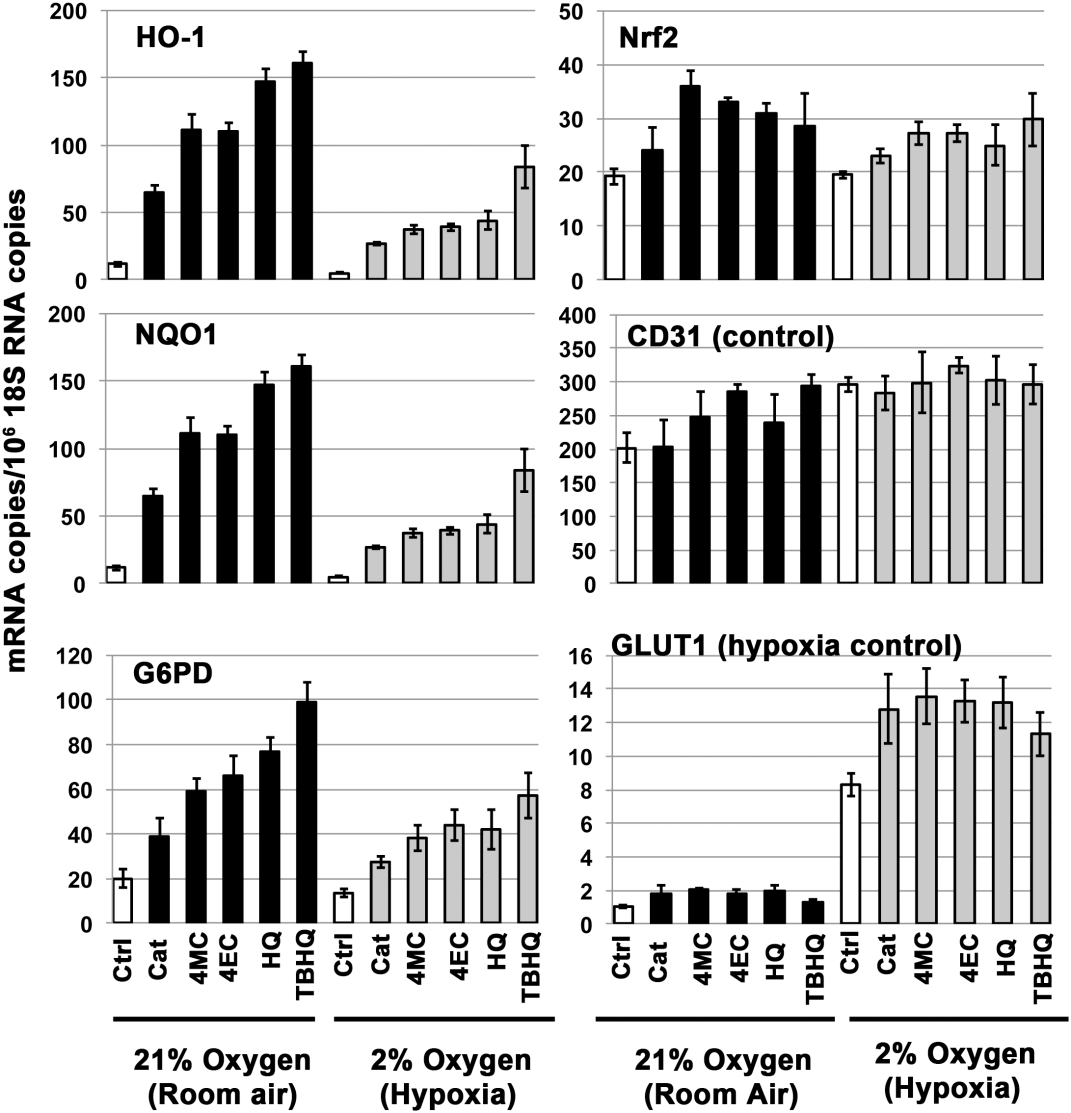
Activation of the Nrf2 pathway by alkyl catechols and catechol is regulated by oxygen. Human microvascular endothelial cells were cultured in 21% oxygen (room air) or 2% oxygen (hypoxia), as indicated, and stimulated with the specified compounds (20 μM each) for 24 hours. RT-PCR, as above, was used to quantify mRNAs; Y-axis = (mRNA copies)/(10^6^ 18S rRNA copies). Cat = catechol, 4MC = 4-methylcatechol, 4EC = 4-ethylcatechol, HQ = hydroquinone, TBHQ = tert-butylhydroquinone (HQ and TBHQ are well known activators of Nrf2, see Fig 1 for chemical structures). Nrf2 target genes = HO-1, NQO1, G6PD; CD31 (PECAM-1) = internal control; GLUT1 (glucose transporter 1) is induced by hypoxia and serves as a positive control for hypoxia-induced gene expression. Error bars = ± S.D.; n ≥ 3 for each data point. ***Summary of data analyses and statistical significance*:** Again, it is important to emphasize that the Nrf2 pathway is activated primarily by stabilization of Nrf2 protein that allows for transcriptional induction of Nrf2 target genes, such as HO-1, NQO1, and G6PD; and therefore, these target gene mRNAs are indicators of Nrf2 pathway activation. Activation of the Nrf2 pathway is not mediated primarily by induction of Nrf2 mRNA, but Nrf2 mRNA induction may contribute modestly as suggested by data shown here in the Nrf2 data panel. Thus, for the HO-1 and NQO1 data sets, representing activation of the Nrf2 pathway, individual comparisons between a specific compound (*i*.*e*. Cat, 4MC, 4EC, HQ, or TBHQ), used at 21% oxygen vs. 2% oxygen, indicated oxygen-dependent differences that are all extremely significant (p<0.001). For the G6PD data set, also representing activation of the Nrf2 pathway, individual comparisons between a specific compound used at 21% oxygen vs. 2% oxygen indicated oxygen-dependent differences that are all very significant (p<0.01) with the exception of Cat (p<0.05, significant). Also, for HO-1 and NQO1, additional comparisons for each of the compounds vs. corresponding Ctrl = extremely significant (p<0.0001) for both 21% oxygen and 2% oxygen. For the G6PD data panel and for both 21% oxygen and 2% oxygen: Ctrl vs. Cat (p<0.005, very significant); Ctrl vs. 4MC, Ctrl vs. 4EC, Ctrl vs. HQ, Ctrl vs. TBHQ (all p<0.0002, extremely significant). For Nrf2, and for both 21% oxygen and 2% oxygen data sets: Ctrl vs. each of the compounds = significant (p≤0.03), with the exception of Cat (21% oxygen) = not significant. For CD31, and for both 21% oxygen and 2% oxygen data sets: Ctrl vs. each of the compounds = not significant, with the exception of Ctrl vs. 4EC and Ctrl vs. TBHQ (21% oxygen) = very significant (p<0.01). Nonetheless, these differences are relatively small in comparison with the large inductions of Nrf2 target gene expression shown in HO-1 and NQO1 data panels. Finally, for the 2% oxygen GLUT1 panel, representing induction of GLUT1 by hypoxia, individual comparisons between Ctrl and each of the compounds indicated differences that were all very significant (p<0.01 to p<0.001). Also, individual comparisons between each experimental group in 2% oxygen with the corresponding experimental group in 21% oxygen indicated differences that were all extremely significant (p<0.0001).

The Nrf2 pathway and its target genes are designed to mitigate the deleterious consequences of oxidative stress, and Nrf2 pathway activation is tightly regulated for this purpose [23, 24]. Indeed, mice with constitutively active Nrf2, due to loss of Keap1, die at young age due to hyperkeratosis [86]. Thus, natural chemical “activators” of the Nrf2 pathway should support Nrf2 target gene expression, not constitutively, but rather in direct relation to threat of oxygen toxicity. To investigate alkyl catechols and catechol, as they relate to Nrf2 pathway activation by oxygen toxicity, we performed parallel experiments with endothelial cells in the presence of 2% oxygen (hypoxia) and 21% oxygen (room air). In addition to 4-ethylcatechol, 4-methylcatechol, and catechol, we tested two well known activators of Nrf2, hydroquinone and tert-butylhydroquinone [51, 78, 87, 88] (see Fig 1 for structures). As shown in Fig 7, all of these compounds robustly induced expression of the Nrf2 target genes, HO-1, NQO1, and G6PD in the presence of 21% oxygen. In the presence of only 2% oxygen, HO-1, NQO1, and G6PD were induced but induction was considerably less than that observed in 21% oxygen. In contrast, the hypoxia-induced gene, glucose transporter 1 (GLUT1), was markedly induced by 2% oxygen, as expected [89]. Thus, these experiments establish that 4-ethylcatechol, 4-methylcatechol, and catechol, each support induction of Nrf2 target gene expression, not constitutively, but rather in co-ordination with the threat of oxygen toxicity. Moreover, each acts similarly to the well known Nrf2 activators, hydroquinone and tert-butylhydroquinone. We did not examine 4-vinylcatechol in this context, but we have no reason to expect that it would differ significantly from the other alkyl catechols. Finally, all of the Nrf2 activators modestly but significantly enhanced GLUT1 mRNA induction by 2% oxygen (Fig 7). Interestingly, reactive oxygen species (ROS) have been reported to depress GLUT1 mRNA in retinal endothelial cells [90]. Thus, activation of the Nrf2 pathway with consequent reduction of ROS may further enhance GLUT1 induction in 2% oxygen. Further studies will be required to elucidate mechanism.

#### Alkyl catechols are biologically active *in vivo*: Induction of Nrf2 target gene expression in mouse kidney and lung

**Fig 8:**
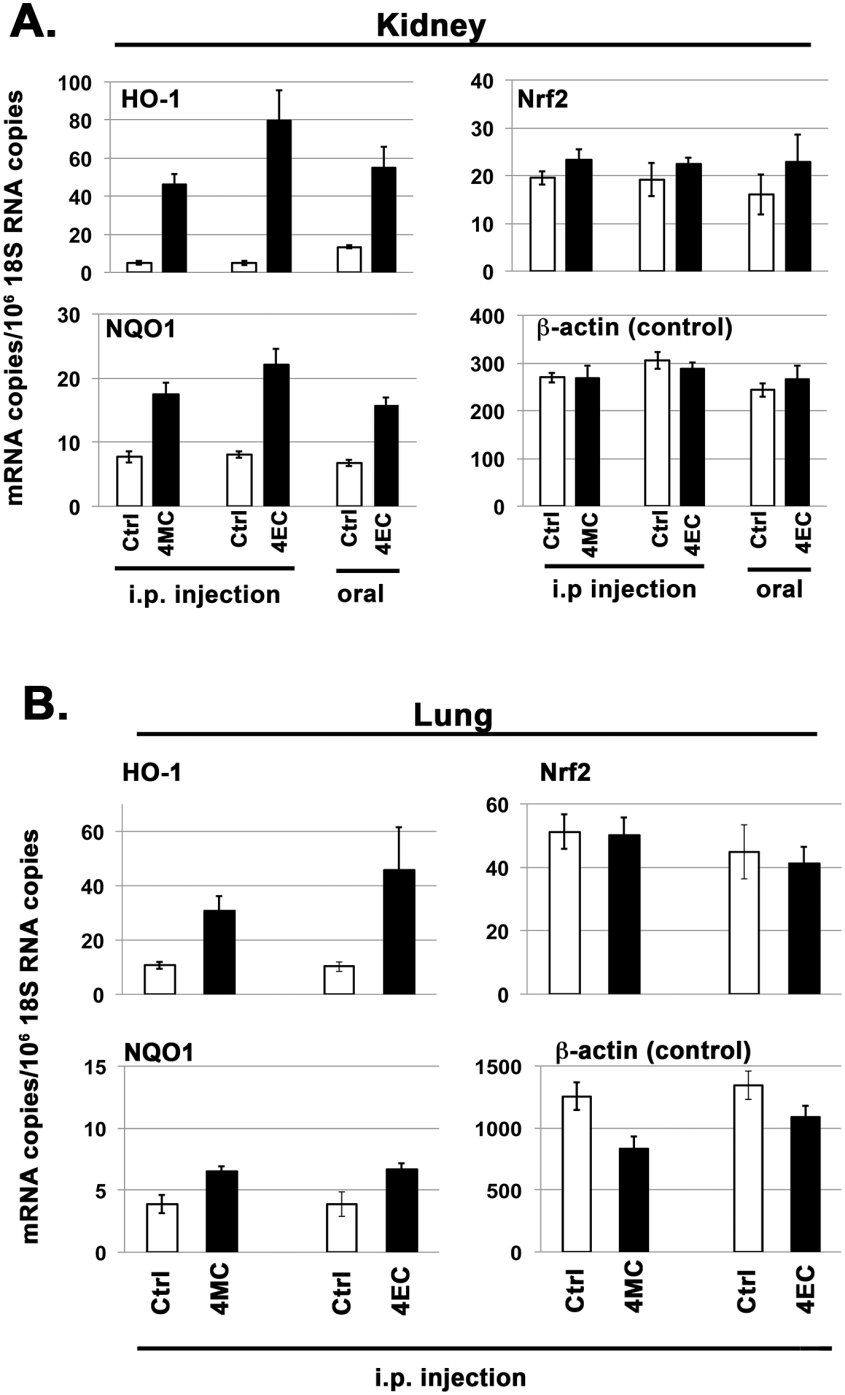
Induction of Nrf2 target genes in mice by 4-methylcatechol and 4-ethylcatechol. RT-PCR analyses; Y-axis = (mRNA copies)/(10^6^ 18S rRNA copies). Error bars = ± S.D.; n ≥ 4 for each data point. **(Panel A, Kidney)** Mice received either intraperitoneal (i.p.) injection or oral gavage of vehicle control (Ctrl), 4-methylcatechol (4MC), or 4-ethylcatechol (4EC) at time zero and again at time zero + 2 hours, for at total dose of 50 mg/kg. Mice were harvested at time zero + 4.5 hours and kidneys dissected for RNA isolation. **(Panel B, Lung)** 4MC or 4EC, as indicated, were administered i.p. at time zero and again time zero + 2 hours for a total of 50 mg/kg. Mice were harvested at time zero + 4.5 hours, and lungs dissected for RNA isolation. ***Summary of data analyses and statistical significance*:** Again, it is important to emphasize that the Nrf2 pathway is activated primarily by stabilization of Nrf2 protein that allows for transcriptional induction of Nrf2 target genes, such as HO-1 and NQO1; and therefore, that robust induction of HO-1 and NQO1 mRNAs, rather than Nrf2 mRNA, indicate Nrf2 pathway activation (see text). (**Panel A)** Inductions of HO-1 and NQO1 mRNAs by 4MC and 4EC, administered either i.p. or orally, were all statistically extremely significant (p < 0.001). For Nrf2: Ctrl vs. 4MC (i.p.) = significant (p<0.05); Ctrl vs. 4EC (i.p.) and Ctrl vs. 4EC (oral) = no significant differences. For β-actin: no statistically significant differences within experimental groups. **(Panel B)** For HO-1, Ctrl vs. 4MC = significant (p = 0.0176), Ctrl vs. 4EC = very significant (p = 0.0025). For NQO1, Ctrl vs. 4MC = significant (p<0.045) and Ctrl vs. 4EC = very significant (p = 0.0031). For Nrf2: Ctrl vs. 4MC and Ctrl vs. 4EC = no significant differences. For β-actin: Ctrl vs. 4MC = very significant (p<0.01) and Ctrl vs. 4EC = significant (p<0.04). Thus, β-actin mRNA was reduced by 4MC and 4EC in lung, in contrast to HO-1 and NQO1 mRNAs that were increased. However, 4MC and 4EC did not reduce β-actin in kidney (Panel A).

Next we investigated induction of Nrf2 target genes HO-1 and NQO1 by 4-methylcatechol and 4-ethylcatechol in mice. For this purpose, and to avoid potentially complicating factors associated with crude rodent diets, mice were placed on the purified rodent diet AIN 76A for 3–4 weeks prior to experiments. Compounds 4-methylcatechol and 4-ethylcatechol were separately administered by intraperitoneal (i.p.) injection, and in some experiments 4-ethylcatechol was administered orally (by gavage). The total dose chosen for each compound (50 mg/kg; ~ 380 μmole /kg) in all of these experiments was less than an oral dose of sulforaphane (150 μmole per 210 gram rat, ~710 μmole /kg) shown previously to induce HO-1 and NQO1 mRNA expression in rat mammary gland [58]. As shown in Fig 8A, i.p. injections of 4-methylcatechol and 4-ethylcatechol each markedly induced expression of HO-1 and NQO1 mRNAs in mouse kidney within 4.5 hours. In addition, oral administration of 4-ethylcatechol markedly induced expression of HO-1 and NQO1 mRNAs in kidney, although less intensely than when administered i.p. We also investigated Nrf2 target gene expression in mouse lung. As shown in Fig 8B, i.p. administrations of 4-methylcatechol and 4-ethylcatechol each significantly induced HO-1 and NQO1 mRNAs in lung within 4.5 hours, although less intensely than in kidney. Thus, similar to findings with primary cell cultures *in vitro*, these alkyl catechols also induce Nrf2 target gene expression in live mice, and induction is readily demonstrable in two major organs (kidney and lung). These experiments also demonstrate that 4-ethylcatechol is potently active when administered orally; and therefore we presume that it enters the bloodstream, in active form, following absorption by the gastrointestinal tract. Finally, we did not investigate 4-vinylcatechol or catechol in these experiments, but based on *in vitro* data (Figs 2 and 3), we expect that they would be active. Nonetheless, it will be important to examine the activity of these compounds *in vivo* in future studies.

### Dietary sources of alkyl catechols and catechol: Biotransformation of inactive dietary phenolic acids to Nrf2 activators by microbial enzymes

#### Generation of the Nrf2 activators 4-vinylcatechol and catechol from inactive dietary phenolics by *Lactobacillus plantarum* and *Lactobacillus brevis*

**Fig 9:**
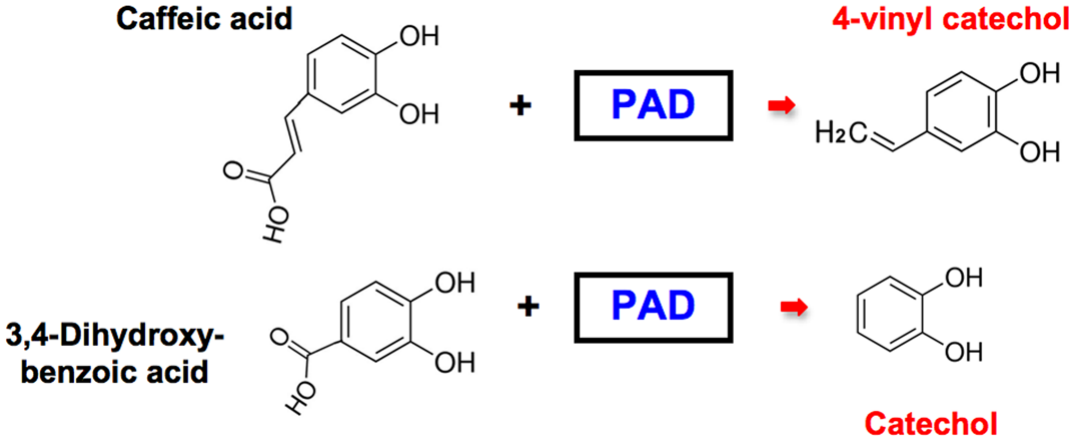
Model for bioconversion of inactive dietary precursors to Nrf2 activators by phenolic acid decarboxylase (PAD). The microbial enzyme, PAD, expressed by *Lactobacillus plantarum*, *Lactobacillus brevis*, and other, but not all, lactobacillus strains convert caffeic acid (inactive) to 4-vinylcatechol (Nrf2 activator). Similarly, PAD converts 3,4-dihydroxybenzoic acid (inactive) to catechol (Nrf2 activator). See text for references and subsequent figures for supporting data.

Findings described above, raise important questions about possible dietary sources of alkyl catechols and catechol. Interestingly, some but not all species of lactobacilli, particularly those associated with traditional fermentation of vegetables and fruits, express the microbial enzyme phenolic acid decarboxylase (PAD) that removes carboxyl groups from phenolic acids [91–93]. In particular, decarboxylation of dietary phenolic acids by microbes expressing PAD suggests a mechanism for generating the Nrf2 activators 4-vinylcatechol and catechol from inactive dietary precursors (see Fig 9). This potentially important and natural mechanism for generating potent activators of the Nrf2 pathway has not been considered previously. Therefore, we performed experiments with specific lactobacilli, known to express PAD, to determine if these lactobacilli can indeed generate Nrf2 activators from inactive dietary precursors.

**Fig 10:**
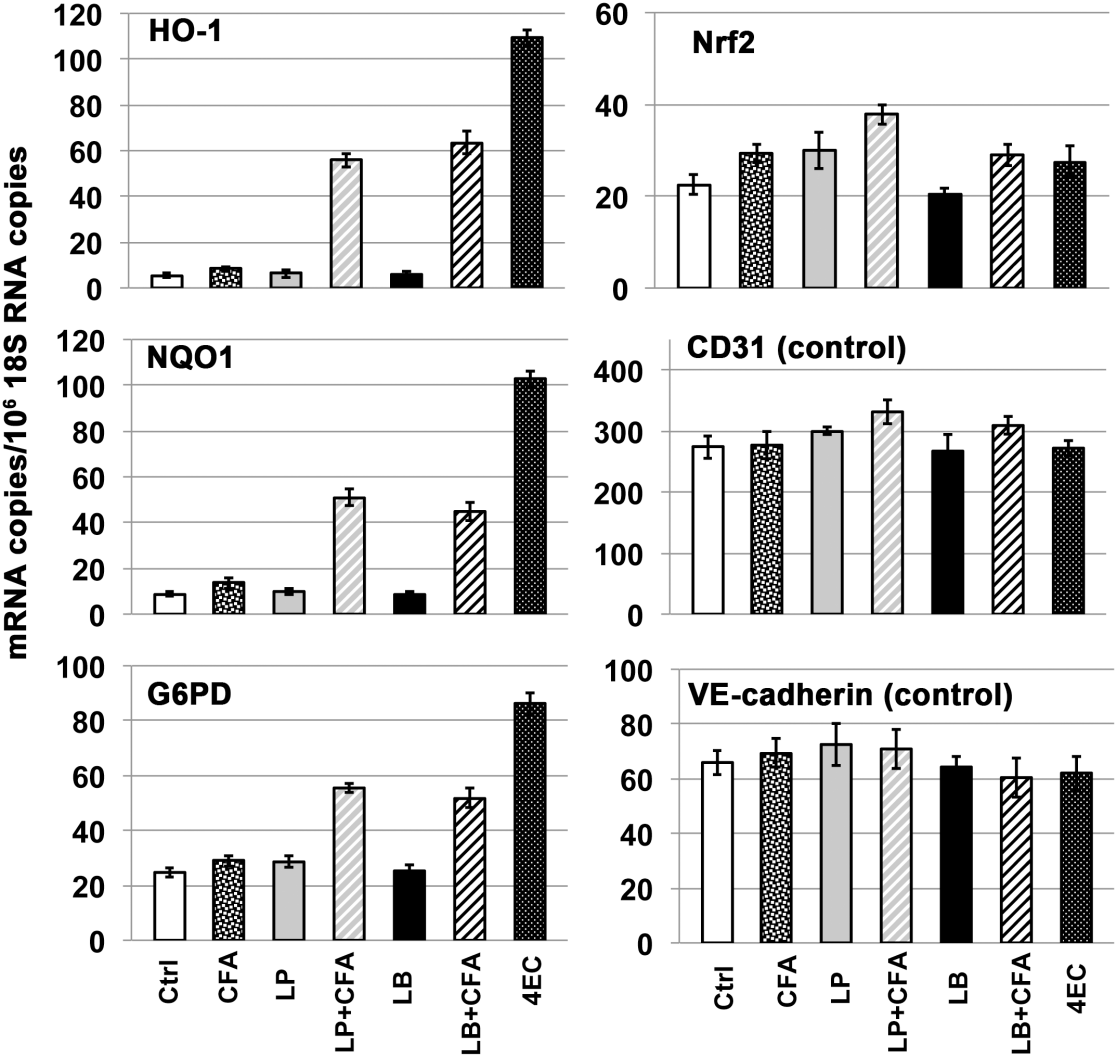
Biotransformation of caffeic acid by *Lactobacillus plantarum* and *Lactobacillus brevis*, as demonstrated with RT-PCR. Y-axis = (mRNA copies)/(10^6^ 18S rRNA copies). Human dermal microvascular endothelial cells, 24 hours after addition of test samples: Ctrl = control, CFA = caffeic acid, LP = control supernatant from *L*. *plantarum* incubated with PBS-glucose and filter-sterilized, (LP + CFA) = supernatant from *L*. *plantarum* incubated with CFA in PBS-glucose and filter-sterilized, LB = control supernatant from *L*. *brevis* incubated with PBS-glucose and filter-sterilized, (LB + CFA) = supernatant from *L*. *brevis* incubated with CFA in PBS-glucose and filter-sterilized. CFA and lactobacillus-incubations with CFA were added to a final concentration corresponding to 30 μM CFA starting material (see Methods). 4EC = 4-ethylcatechol positive control (30 μM). Nrf2 target genes = HO-1, NQO1, G6PD. Control mRNAs = CD31 and VE-cadherin. Error bars = ± S.D.; n ≥ 3 for each data point. ***Summary of data analyses and statistical significance*:** As described in previous figures and in the text, induction of the Nrf2 target genes HO-1, NQO1, and G6PD, rather than induction of Nrf2 mRNA, indicates activation of the Nrf2 pathway. Only LP+CFA, LB+CFA, and 4EC (positive control) demonstrated Nrf2 pathway activation by these criteria. For HO-1, NQO1, and G6PD data panels, individual comparisons between Ctrl (or CFA) vs. LP+CFA, Ctrl (or CFA) vs. LB+CFA, and Ctrl (or CFA) vs. 4EC indicated differences that are all extremely significant (p< 0.0001). In contrast, for the HO-1, NQO1, and G6PD panels, individual comparisons between Ctrl vs. CFA, Ctrl vs. LP, and Ctrl vs. LB indicated no significant differences. For Nrf2, Ctrl vs. CFA = small but significant difference (p<0.05), Ctrl vs. LP = not significant, Ctrl vs. LP+CFA = very significant (p<0.01), Ctrl vs. LB = not significant, Ctrl vs. LB+CFA = significant (p<0.05), Ctrl vs. 4EC = significant (p<0.05). Finally, for CD31 and VE-cadherin data sets, statistical analyses indicated no significant differences.

**Fig 11:**
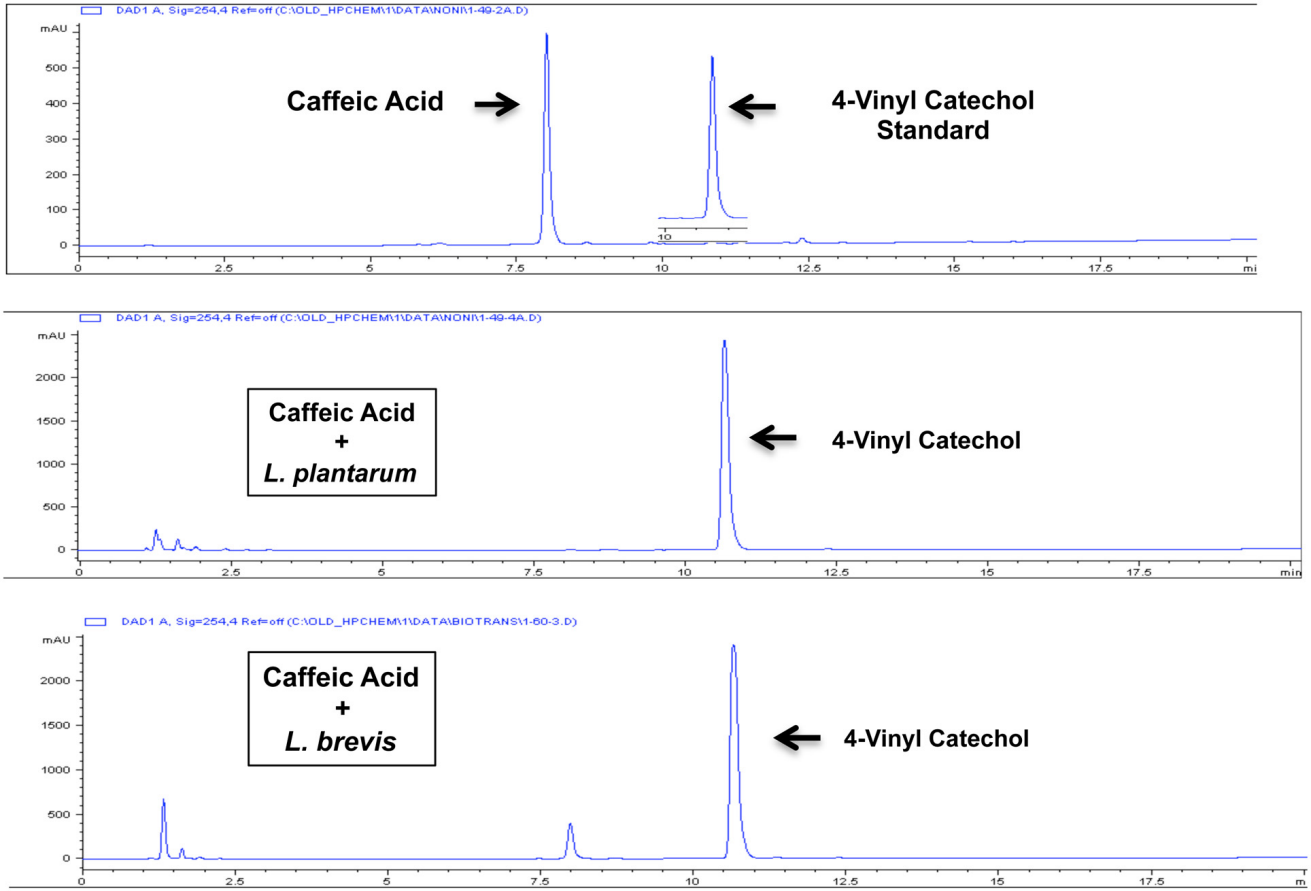
Biotransformation of caffeic acid by *L*. *plantarum* and *L*. *brevis*, as demonstrated with HPLC. Y-axis = absorbance at 254nm (mAU), X-axis = minutes. Top panel: HPLC of caffeic acid and 4-vinylcatechol standards. Middle panel: HPLC of supernatant from caffeic acid + *L*. *plantarum* incubation, consistent with conversion of caffeic acid to 4-vinylcatechol. Bottom panel: HPLC of supernatant from caffeic acid + *L*. *brevis* incubation, consistent with conversion of caffeic acid to 4-vinylcatechol. Retention times: caffeic acid = 8.1 minutes, 4-vinylcatechol = 10.7 minutes.

We began with *Lactobacillus plantarum* and *Lactobacillus brevis*, both of which are found in traditional vegetable, fruit, and malt whiskey fermentations and both of which express PAD [91–95]. We incubated *L*. *plantarum* and *L*. *brevis* with caffeic acid that is a common hydroxycinnamic acid found in all plants and in various foods including fruits, vegetables, nuts, grains, spices, and coffee [96]. After overnight incubation (see Methods), supernatants were collected by centrifugation, sterilized by filtration, and added to human endothelial cells, at equal dilution so that the final caffeic acid concentration was 30 μM. After 24 hours, cells were harvested and RNA isolated to measure expression of Nrf2 target genes. As shown in Fig 10, caffeic acid (CFA) alone and control supernatants from *L*. *plantarum* or *L*. *brevis* had no significant effect on induction of Nrf2 target genes HO-1, NQO1, or G6PD. In contrast, supernatants from incubations containing *L*. *plantarum* + caffeic acid and *L*. *brevis* + caffeic acid strongly induced all three Nrf2 target genes. Thus, as predicted by the model presented in Fig 9, these experiments clearly indicate that select strains of lactobacilli expressing PAD can generate potent Nrf2 activator from inactive precursor. As expected, chemical analyses with high-pressure liquid chromatography (HPLC) demonstrated nearly complete conversion of caffeic acid by *L*. *plantarum* and *L*. *brevis* to a compound with chromatographic retention time identical to 4-vinylcatechol (Fig 11). Thus, these experiments indicate that *L*. *plantarum* and *L*. *brevis* can each biotransform caffeic acid to potently activate the Nrf2 pathway.

**Fig 12:**
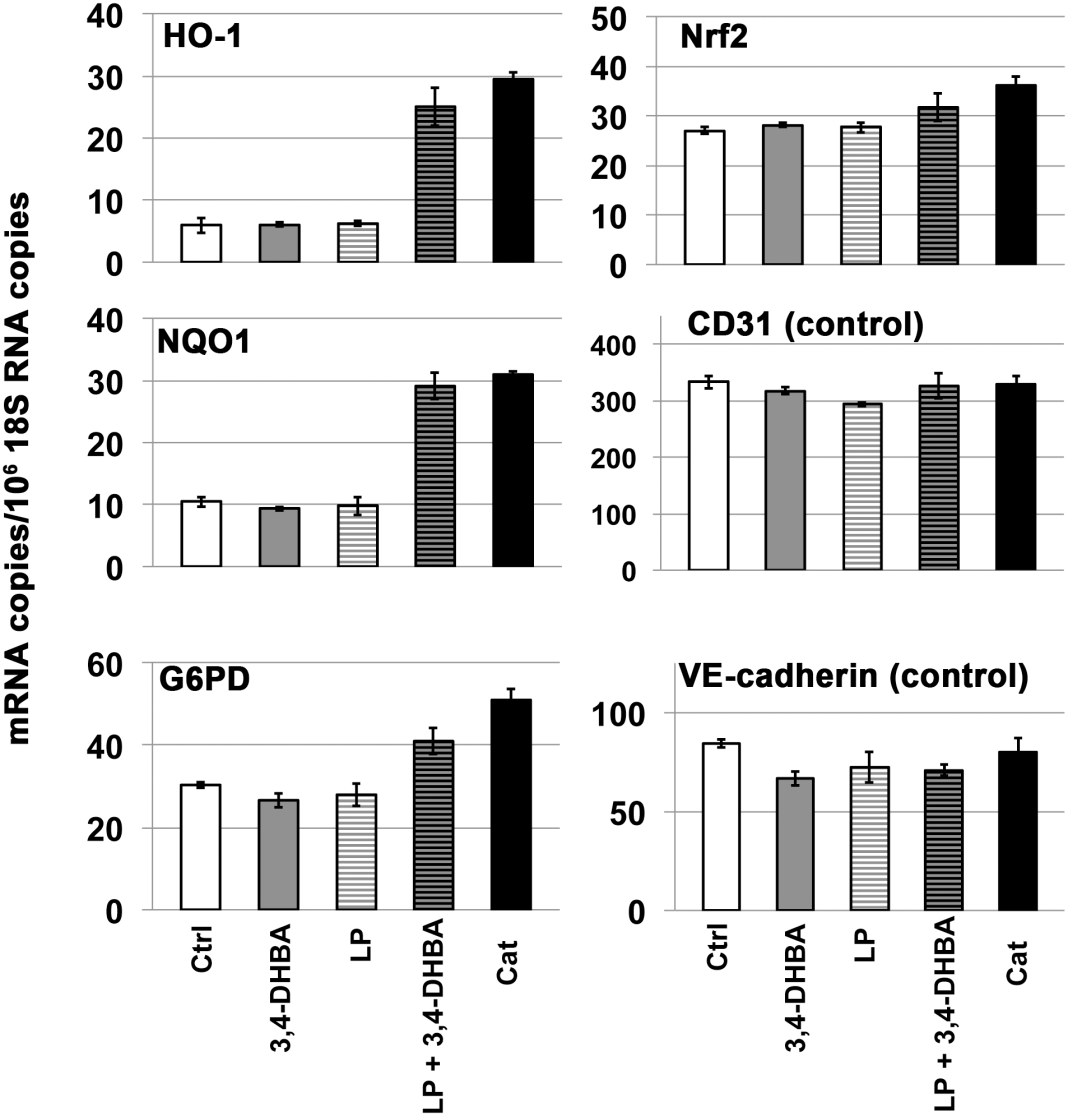
Biotransformation of 3,4-dihydroxybenzoic acid by *Lactobacillus plantarum*, as demonstrated with RT-PCR. Y-axis = (mRNA copies)/(10^6^ 18S rRNA copies). Human dermal microvascular endothelial cells, 24 hours after addition of test samples: Ctrl = control, 3,4-DHBA = 3,4-dihydroxybenzoic acid, LP = control supernatant from *L*. *plantarum* incubated with PBS-glucose and filter-sterilized, (LP + 3,4-DHBA) = supernatant from *L*. *plantarum* incubated with 3,4-DHBA in PBS-glucose and filter-sterilized. 3,4-DHBA and lactobacillus-incubations with 3,4-DHBA were added to a final concentration corresponding to 30 μM 3,4-DHBA starting material (see Methods). Cat = catechol positive control (30 μM). Nrf2 target genes = HO-1, NQO1, G6PD. Control mRNAs = CD31 and VE-cadherin. Error bars = ± S.D.; n ≥ 3 for each data point. ***Summary of data analyses and statistical significance*:** As described in previous figures and in the text, induction of the Nrf2 target genes HO-1, NQO1, and G6PD, rather than induction of Nrf2 mRNA, indicates activation of the Nrf2 pathway. For HO-1 and NQO1 data panels, individual comparisons for Ctrl (or 3,4-DHBA) vs. LP+3,4-DHBA and Ctrl (or 3,4-DHBA) vs. Cat indicated differences that are extremely statistically significant (p< 0.0001). For the G6PD data panel, individual comparisons for Ctrl vs. LP+3,4-DHBA and Ctrl vs. Cat indicated differences that are also extremely significant (p< 0.0005). In contrast, for the HO-1, NQO1, and G6PD panels, individual comparisons for Ctrl vs. 3,4-DHBA and Ctrl vs. LP indicated no significant differences. For Nrf2, only Ctrl vs. Cat was statistically significant (p<0.05). For CD31: no statistically significant differences. For VE-cadherin, only Ctrl vs. 3,4-DHBA was statistically significant (p<0.05).

**Fig 13:**
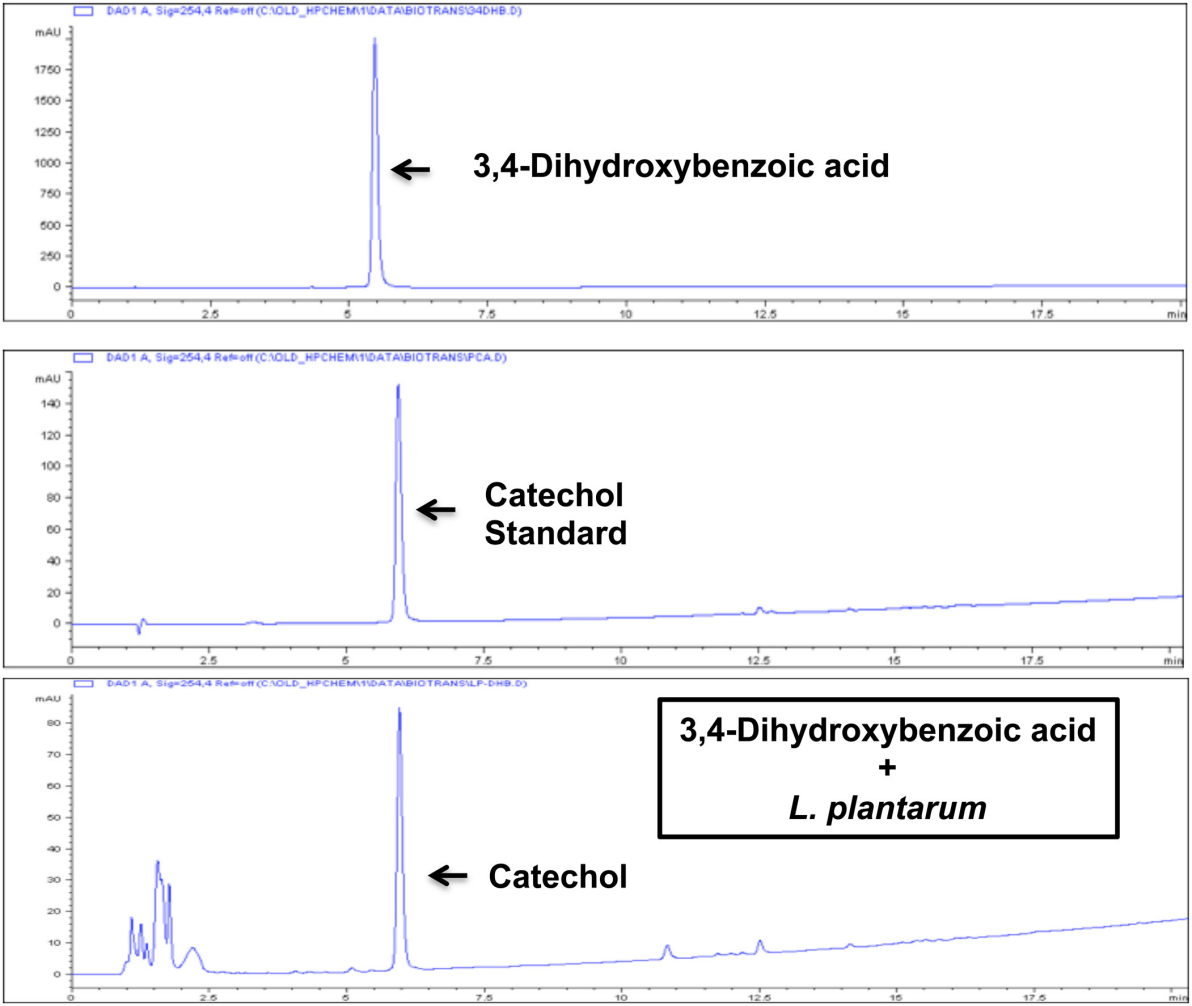
Biotransformation of 3,4-dihydroxybenzoic acid by *L*. *plantarum*, as demonstrated with HPLC. Y-axis = absorbance at 254nm (mAU), X-axis = minutes. Top panel: HPLC of 3,4-dihydroxybenzoic acid standard. Middle panel: HPLC of catechol standard. Bottom panel: HPLC of supernatant from 3,4-dihydroxybenzoic acid + *L*. *plantarum* incubation, consistent with conversion of 3,4-dihydroxybenzoic acid to catechol. Retention times: 3,4-dihydroxybenzoic acid = 5.5 minutes, catechol = 6.0 minutes.

Next, in experiments similar to those with caffeic acid (above), we examined biotransformation of 3,4-dihydroxybenzoic acid (3,4-DHBA), by *L*. *plantarum*. 3,4-DHBA, also known as protocatechuic acid, is found in brightly colored berries [96]. 3,4-DHBA also is a prominent dietary intermediate in the metabolism of anthocyanins found in these same foods [97, 98]. As shown in Fig 12, neither 3,4-DHBA nor supernatant from *L*. *plantarum* alone had any significant effect on induction of Nrf2 target genes HO-1, NQO1, or G6PD. In contrast, supernatant from the incubation of *L*. *plantarum* + 3,4-DHBA induced all three Nrf2 target genes comparably to catechol. Moreover, HPLC demonstrated that *L*. *plantarum* converts 3,4-DHBA to a compound with chromatographic retention time identical to catechol (Fig 13). Thus, these experiments indicate that *L*. *plantarum* can decarboxylate 3,4-DHBA to produce catechol, thereby generating yet another Nrf2 activator.

**Fig 14:**
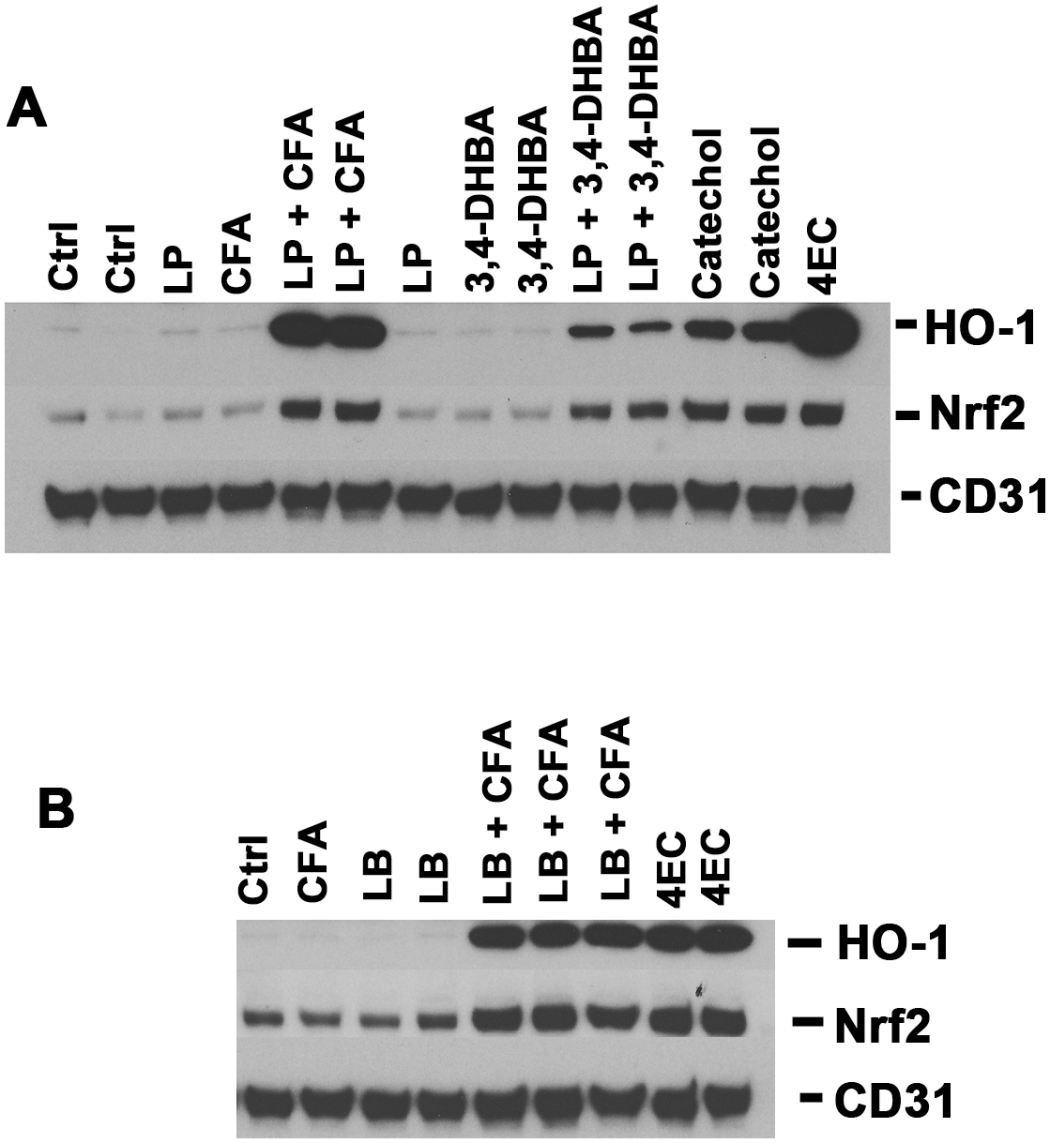
Biotransformation of caffeic acid and 3,4-dihydroxybenzoic acid, as demonstrated with western blotting. (A) Biotransformation by *L*. *plantarum* as demonstrated with western blotting of human dermal microvascular endothelial cells, harvested 24 hours after addition of test samples. Blots were stained for the Nrf2 target gene HO-1, Nrf2 itself, and CD31 as loading control. Key: Ctrl = control, CFA = caffeic acid, LP = control supernatant from *L*. *plantarum* incubated with PBS-glucose and filter-sterilized, (LP + CFA) = supernatant from *L*. *plantarum* incubated with CFA in PBS-glucose and filter-sterilized, 3,4-DHBA = 3,4-dihydroxybenzoic acid, (LP + 3,4-DHBA) = supernatant from *L*. *plantarum* + 3,4-DHBA incubated in PBS-glucose and filter-sterilized. CFA, 3,4-DHBA, and lactobacillus-incubations with each were added to a final concentration corresponding to 30 μM CFA and 30 μM 3,4-DHBA starting material (see Methods). Positive controls = catechol (30 μM) and 4-ethylcatechol (4EC, 30 μM). (B) Biotransformation by *L*. *brevis* (LB), with experimental conditions otherwise identical to panel (A), above. Also, for experiment shown in panel (B), 4EC = 4-ethylcatechol positive control was added to a final concentration of 15 μM instead of 30 μM.

In additional experiments, we independently confirmed these findings with western blot analyses of HO-1 protein expression. As shown in Fig 14A, supernatants from incubations of *L*. *plantarum* + caffeic acid (CFA) strongly induced HO-1 protein whereas CFA alone and supernatants from *L*. *plantarum* alone were without effect. Although less potent than supernatants from *L*. *plantarum* + caffeic acid, supernatants from *L*. *plantarum* + 3,4-DHBA also induced HO-1 protein whereas 3,4-DHBA alone did not induce HO-1 detectably above baseline. In addition to increasing HO-1 protein, incubations from *L*. *plantarum* + CFA and *L*. *plantarum* + 3,4-DHBA also consistently increased Nrf2 protein, as quantified with Image J (> 2-fold for *L*. *plantarum* + 3,4-DHBA; > 3-fold for *L*. *plantarum* + CFA). Similar findings of increased Nrf2 protein were made with experiments presented in Fig 3. Again, such increase is most likely attributable to increased Nrf2 protein stabilization due to release of Nrf2 from Keap1 and thereby reduced proteosomal degradation of Nrf2, rather than an increase in Nrf2 mRNA copy number (compare Nrf2 protein in Fig 14A with Nrf2 mRNA copy numbers in Figs 10 and 12). Nonetheless, some contribution due to increase in Nrf2 copy number is plausible; and, as discussed previously, the Nrf2 promoter contains antioxidant response element sequences [77]. Regardless, all of these data are consistent with activation of the Nrf2 pathway by supernatants from *L*. *plantarum* + CFA and *L*. *plantarum* + 3,4-DHBA. Also, as shown in Fig 14B, western blotting analyses of experiments with supernatants from *L*. *brevis* + CFA demonstrated findings comparable to those made with *L*. *plantarum* + CFA, consistent with the RT-PCR data and HPLC analyses presented in Figs 10 and 11. Finally, findings here suggest important experiments for future work, such as investigations on activation of the Nrf2 pathway *in vivo* by supernatants from incubations containing *L*. *plantarum* + CFA or *L*. *brevis* + CFA, and also by diets containing CFA together with *L*. *plantarum* or *L*. *brevis*.

#### Multi-enzyme conversion of inactive dietary phenolics to the Nrf2 activator 4-ethylcatechol by *Lactobacillus collinoides*

**Fig 15:**
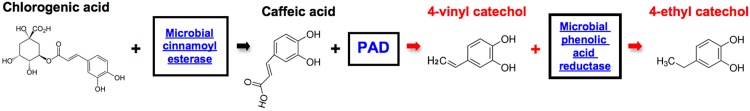
Model for multi-step bioconversion of inactive dietary precursors to Nrf2 activators by *Lactobacillus collinoides*. Microbial cinnamoyl esterase from *L*. *collinoides* converts chlorogenic acid (inactive) to caffeic acid (inactive), thereby providing substrate for phenolic acid decarboxylase (PAD)-mediated generation of 4-vinyl catechol (Nrf2 activator). Finally, microbial phenolic acid reductase, also expressed by *L*. *collinoides*, reduces 4-vinylcatechol to 4-ethylcatechol (Nrf2 activator). See text for supporting references and subsequent figures for supporting data.

**Fig 16:**
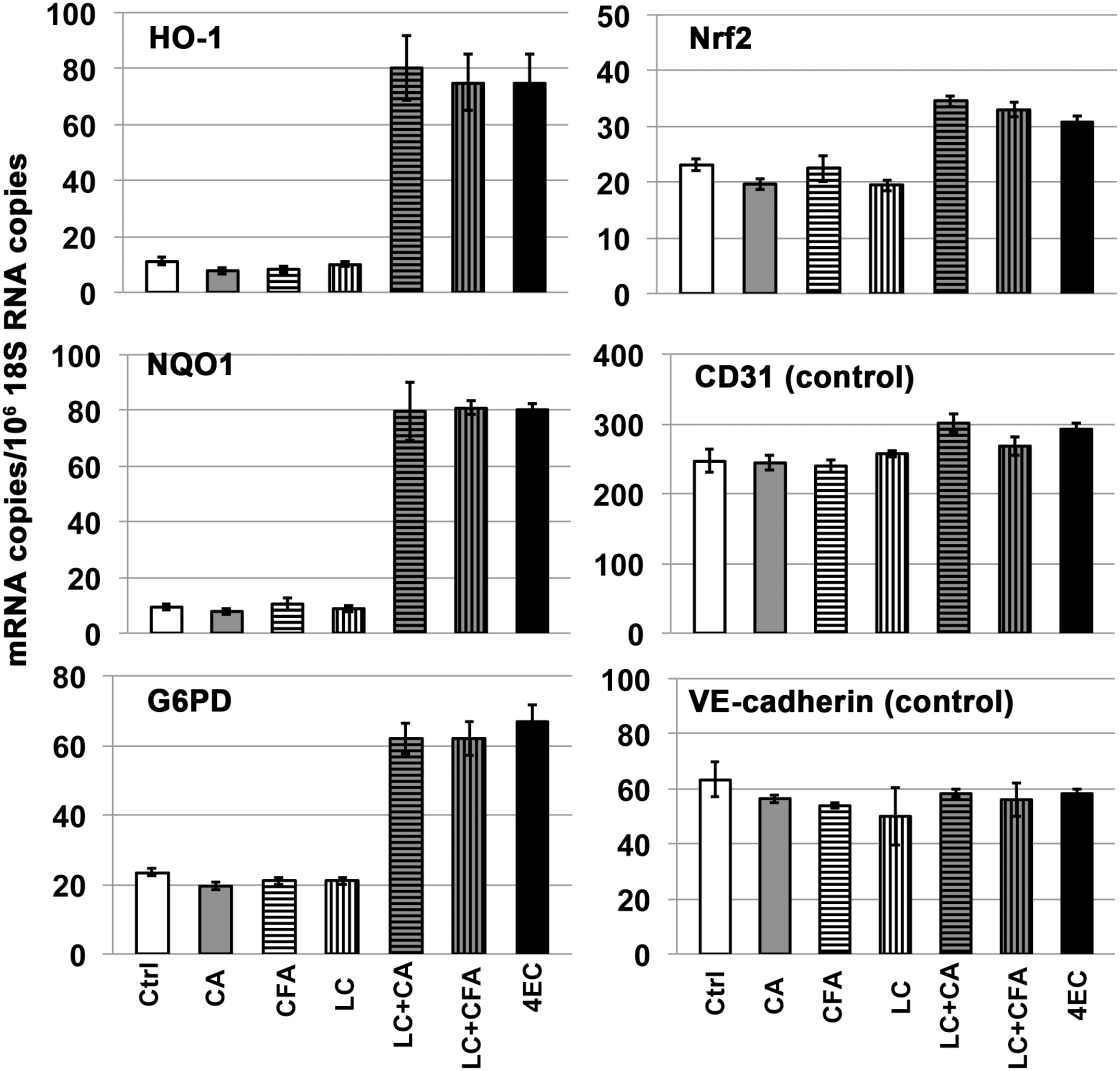
Biotransformation of chlorogenic acid and caffeic acid by *Lactobacillus collinoides*, as measured with RT-PCR. Y-axis = (mRNA copies)/(10^6^ 18S rRNA copies). RNA was isolated from human dermal microvascular endothelial cells, 24 hours after addition of test samples: Ctrl = control, CA = chlorogenic acid, CFA = caffeic acid, LC = control supernatant from *L*. *collinoides* incubated with PBS-glucose and filter-sterilized, (LC + CA) = supernatant from *L*. *collinoides* incubated with CA in PBS-glucose and filter-sterilized, (LC + CFA) = supernatant from *L*. *collinoides* incubated with CFA in PBS-glucose and filter-sterilized. CA, CFA, and *L*. *collinoides* incubations with each were added to a final concentration corresponding to 30 μM CA and 30 μM CFA starting material (see Methods). (4EC) = 4-ethylcatechol positive control (30 μM). Nrf2 target genes = HO-1, NQO1, G6PD; control mRNAs = CD31 and VE-cadherin. Error bars = ± S.D.; n ≥ 3 for each data point. ***Summary of data analyses and statistical significance*:** As discussed previously, the Nrf2 pathway is activated primarily by stabilization of Nrf2 protein that allows for transcriptional induction of Nrf2 target genes, such as HO-1, NQO1, and G6PD; and therefore, these target gene mRNAs are indicators of Nrf2 pathway activation. Activation of the Nrf2 pathway is not mediated primarily by induction of Nrf2 mRNA, but Nrf2 mRNA induction may contribute modestly as suggested by data shown here (see text for further explanation and references). Thus, for HO-1, NQO1 and G6PD data panels, individual comparisons for Ctrl vs. LC+CA, Ctrl vs. LC+CFA, and Ctrl vs. 4EC indicated differences that are all extremely statistically significant (p< 0.0001). In contrast, individual comparisons for Ctrl vs. CA, Ctrl vs. CFA, and Ctrl vs. LC indicated no significant differences. For Nrf2, Ctrl vs. CA, Ctrl vs. CFA, and Ctr vs. LC = no significant differences; Ctrl vs. LC+CA, Ctrl vs. LC+CFA, and Ctrl vs. 4EC = all very significant differences (p<0.01). For CD31 and VE-cadherin: no statistically significant differences.

**Fig 17:**
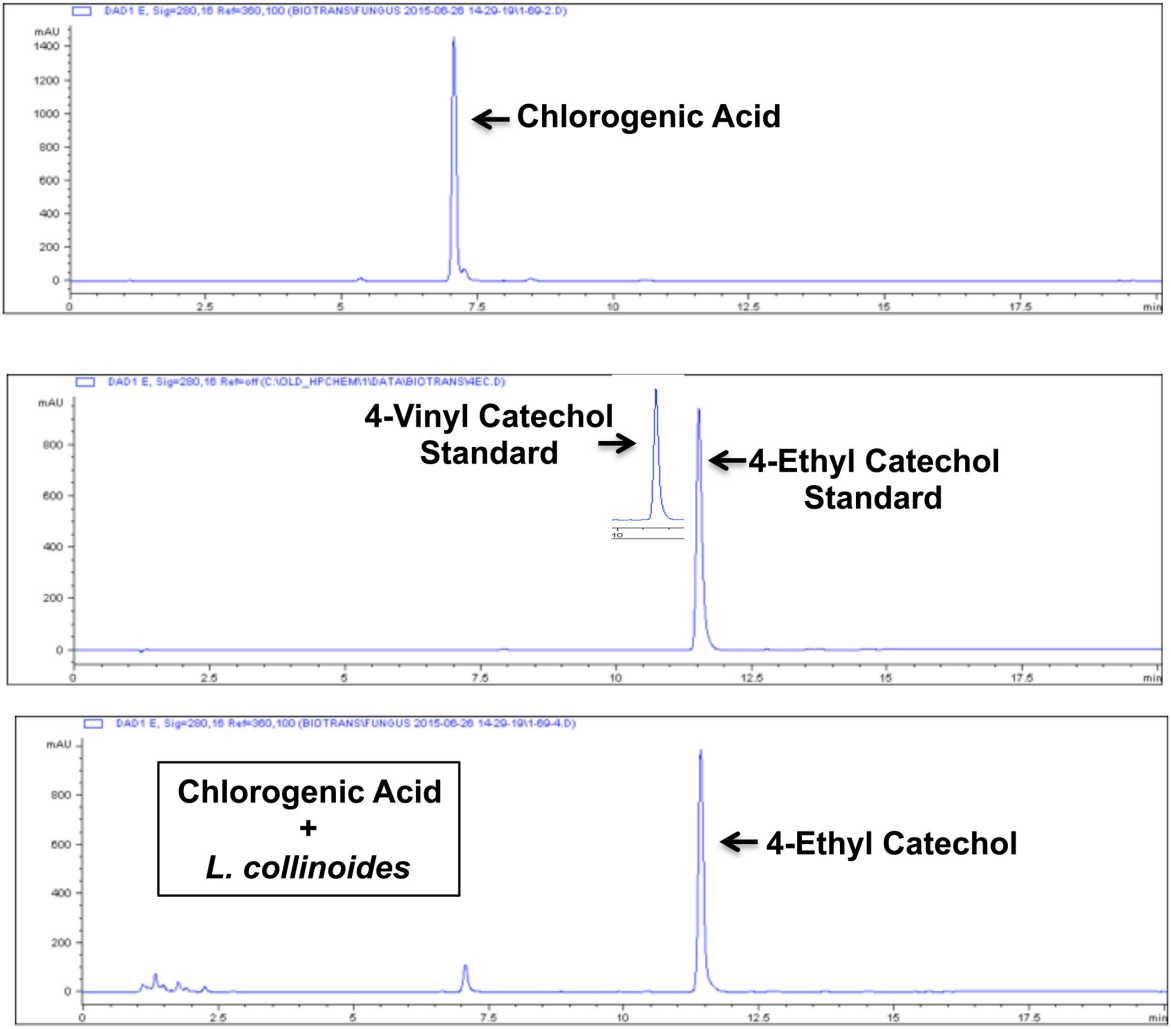
Biotransformation of chlorogenic acid by *L*. *collinoides*, as demonstrated with HPLC. Y-axis = absorbance at 254nm (mAU), X-axis = minutes. Top panel: HPLC of chlorogenic acid standard. Middle panel: HPLC of 4-vinylcatechol and 4-ethylcatechol standards. Bottom panel: HPLC of supernatant from chlorogenic acid + *L*. *collinoides* incubation, consistent with conversion of chlorogenic acid to 4-ethylcatechol. Retention times: chlorogenic acid = 7.1 minutes, 4-vinylcatechol = 10.7 minutes, 4-ethylcatechol = 11.6 minutes.

**Fig 18:**
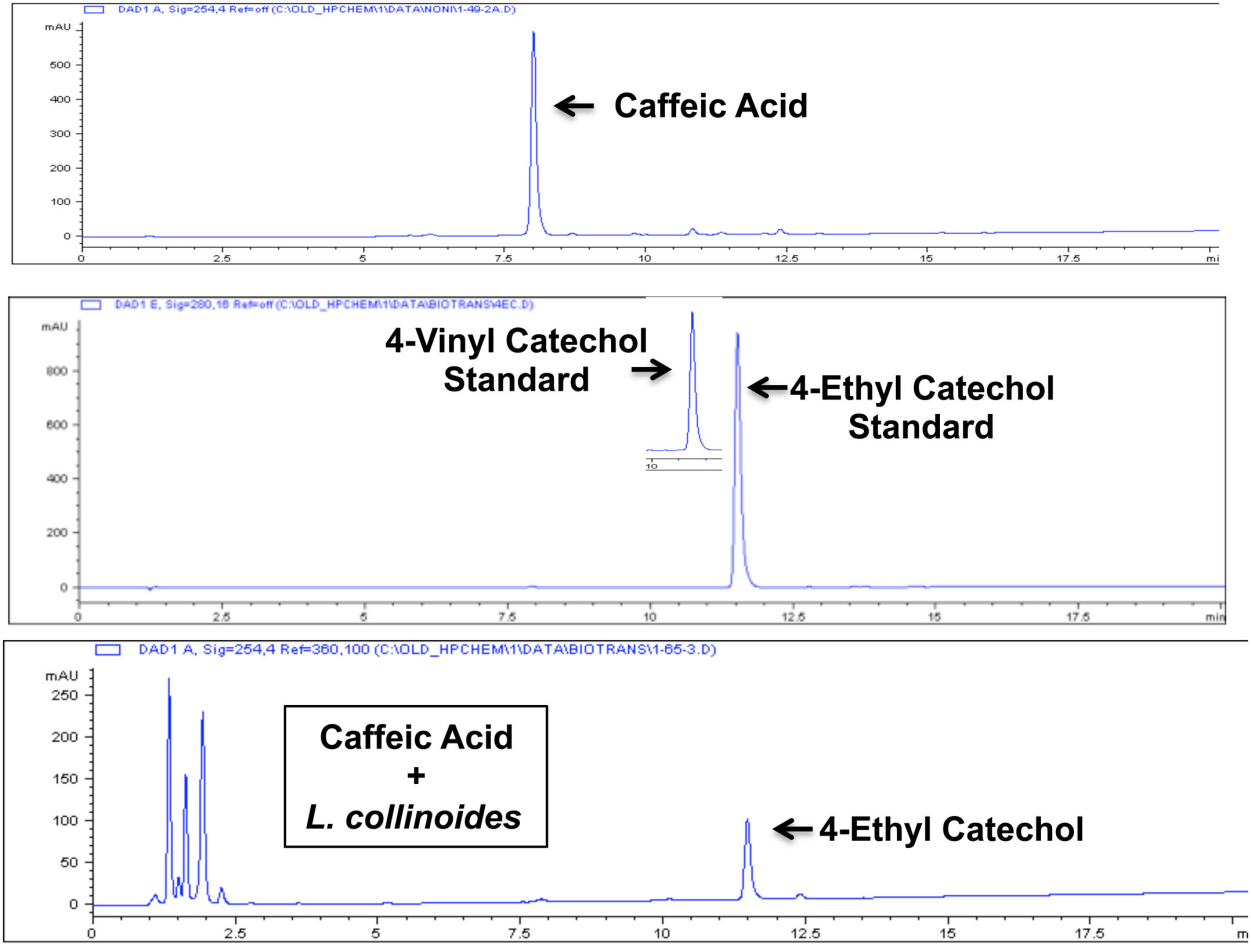
Biotransformation of caffeic acid by *L*. *collinoides*, as demonstrated with HPLC. Y-axis = absorbance at 254nm (mAU), X-axis = minutes. Top panel: HPLC of caffeic acid standard. Middle panel: HPLC of 4-vinylcatechol and 4-ethylcatechol standards. Bottom panel: HPLC of supernatant from caffeic acid + *L*. *collinoides* incubation, consistent with bioconversion of caffeic acid to 4-ethylcatechol. Retention times: caffeic acid = 8.1 minutes, 4-vinylcatechol = 10.7 minutes, 4-ethylcatchol = 11.6 minutes.

Other potential food sources of alkyl catechols are suggested by the chemical composition of traditional ciders, fermented by *Lactobacillus collinoides* [99–101]. Similarly to *L*. *plantarum* and *L*. *brevis*, *L*. *collinoides* also has been shown to express PAD [100]; and, *L*. *collinoides* also expresses cinnamoyl esterase [100] that cleaves chlorogenic acid to caffeic acid (Fig 15). Chlorogenic acid is an ester of caffeic acid with quinic acid, and it is found in a wide range of fruits and vegetables and in particularly high concentrations in coffee [102]. Consequently, chlorogenic acid is common dietary substance and a potentially important precursor to alkyl catechols. In addition to PAD and cinnamoyl esterase that, together, can convert chlorogenic acid to 4-vinylcatechol (Fig 15), *L*. *collinoides* also expresses phenolic acid reductase (vinylphenol reductase) [100] that can reduce 4-vinylcatechol to 4-ethylcatechol. Thus, the *L*. *collinoides* enzymes PAD, cinnamoyl esterase, and phenolic acid reductase have the potential in combination to generate the Nrf2 activator 4-ethylcatechol from both chlorogenic acid and caffeic acid. To test this possibility, we performed incubations with *L*. *collinoides* and chlorogenic acid and caffeic acid, comparable to incubations performed with *L*. *plantarum* and *L*. *brevis* (above). As shown in Fig 16, chlorogenic acid, caffeic acid, and supernatant from *L*. *collinoides* alone showed no significant induction of Nrf2 target genes HO-1, NQO1, or G6PD. In contrast, supernatants from incubations of *L*. *collinoides* + chlorogenic acid and *L*. *collinoides* + caffeic acid strongly induced all three Nrf2 target genes comparably to 4-ethylcatechol (4EC). Consistent with these findings, HPLC demonstrated nearly complete conversion of chlorogenic acid by *L*. *collinoides* to a compound with chromatographic retention time identical to 4-ethylcatechol but not 4-vinylcatechol (Fig 17). Similarly, HPLC demonstrated conversion of caffeic acid by *L*. *collinoides* to a compound with chromatographic retention time identical to 4-ethylcatechol (Fig 18). Thus, these experiments support the model presented in Fig 15 wherein multiple enzymes of *L*. *collinoides*, convert both chlorogenic acid and caffeic acid to 4-ethylcatechol, a potent Nrf2 activator. In contrast, *L*. *plantarum* and *L*. *brevis* express PAD but apparently lack phenolic acid reductase, and thereby convert caffeic acid to 4-vinylcatechol but fail to reduce it to 4-ethylcatechol.

#### Lactobacillus species that do not biotransform caffeic acid to Nrf2 activator

In contrast to our findings with *L*. *plantarum*, *L*. *brevis*, and *L*. *collinoides* described above, we found in identical experiments that three other strains of lactobacillus did not biotransform caffeic acid to Nrf2 activator, as measured with RT-PCR for Nrf2 target genes HO-1, NQO1, and G6PD (S4 Fig). These strains, all from the American Type Culture Collection (ATCC) are: *L*. *reuteri* MM4-1A isolated from human mother’s milk (ATCC PTA-6475), *L*. *ruminus* isolated from bovine rumen (ATCC 27780), and *L*. *paracaseii* (ATCC 25302), associated with fermentation of dairy products. Presumably, these strains of lactobacilli did not biotransform caffeic acid because they do not express PAD significantly; however, we did not investigate PAD directly. Interestingly, none of these strains are typically associated with plant sources [103–105]. In contrast, *L*. *plantarum*, *L*. *brevis*, and *L*. *collinoides*, all of which express PAD, are typically associated with plant fermentations [99, 106], suggesting a logical connection between bacterial expression of PAD and natural co-residence with plants that produce phenolic acids.

## Discussion

The Nrf2 cell defense pathway provides natural protection against oxidative stress and chemical toxicity through the induction of numerous target genes [23, 24, 27]. However, robust activation of Nrf2-mediated protection requires not only reactive oxygen species (ROS) but also relatively small electrochemical co-factors, most often referred to as “chemical inducers” or “Nrf2 activators”. These “inducers” or “Nrf2 activators” amplify the effects of ROS by cycling through oxidation-reduction reactions [51], and ultimately by modifying sulfhydryl groups to liberate Nrf2 from negative control by Keap1 [31, 32]. Data presented here illustrate that the alkyl catechols, 4-ethylcatechol, 4-vinylcatechol, and 4-methycatechol, are potently active and natural Nrf2 co-factors in human endothelial cells (Figs 2, 3 and 6), human astrocytes (Figs 2 and 6), human keratinocytes (Fig 2), and mouse kidney and lung (Fig 8) without any evidence of cytotoxicity (S1 Fig). These cell types and tissues have all been implicated in disease processes involving oxidative stress; and, consequently, our findings have potentially broad implications for preventing a variety of common diseases and for reducing the consequences of aging, particularly cancer and neurodegenerative disorders (reviewed in the Introduction). In addition to potently supporting expression of Nrf2 target genes, the akyl catechols and catechol increase Nrf2 protein as determined with western blotting (Fig 3), increase nuclear concentration of Nrf2 as determined with immunohistochemistry (Fig 4), and protect cells from hydrogen peroxide-induced cell death (S2 Fig). Also, as expected for naturally effective Nrf2 co-factors, the alkyl catechols and catechol support activation of the Nrf2 pathway in conjunction with the threat of oxygen toxicity and do not activate the Nrf2 pathway constitutively (Fig 7). This is important because the Nrf2 pathway is tightly regulated to control oxidative stress [23, 24]; and constitutive, unregulated activation of Nrf2 can have deleterious consequences [86].

Although catechol was among the first compounds recognized as a cancer-protective compound [51], 4-ethylcatechol, 4-vinylcatechol, and 4-methylcatechol had not received attention. Nonetheless, these alkyl catechols each satisfy the well-defined and relatively strict structural criteria for “oxidation-reduction lability” that is required for a compound to induce cancer protective enzymes [51, 78]. Also, we found that these alkyl catechols are more potent than catechol and comparably potent to sulforaphane, a well-characterized Nrf2 inducer. In contrast, we found that a variety of other phenolic compounds did not support the Nrf2 pathway significantly in our assays (summarized in Fig 5). Thus, apart from sulforaphane, we believe that the alkyl catechols may be the most potent of the naturally occurring Nrf2 co-factors. Moreover, the relatively small size and simple structure of these compounds, in combination with our findings in experiments involving mice (Fig 8), suggest the possibility of better bioavailability.

An important and unresolved question is whether or not the Nrf2 pathway evolved independently of exogenous “inducers” or “activators” or, instead, evolved in the presence of ubiquitously available, redox-cycling, electrochemicals that “collaborate” with ROS to activate Nrf2. If the latter is true, exogenous Nrf2 “inducers” should be considered more appropriately as co-factors that are essential to support proper function of the Nrf2 pathway. From both evolutionary and biochemical perspectives, it seems likely that the Nrf2 pathway evolved in the presence of electrochemical co-factors. However, the identity of such co-factors has remained obscure. Few natural compounds truly satisfy the structural criteria for “oxidation-reduction lability” [51, 78]. Among those that qualify, sulforaphane [53] is found only in cruciferous vegetables; another well-characterized and natural Nrf2 inducer, curcumin [55, 63, 107], is limited to a few sources such as turmeric; and, similarly, hydroquinone [51] is rare in natural sources. Other well-characterized Nrf2 inducers with appropriate redox cycling characteristics, such as tert-butylhdroquinone [51], are not found naturally. By contrast, there are very numerous and diverse natural dietary sources of alkyl catechols and catechol, as described below. The abundant natural sources of alkyl catechols and catechol, together with chemical structure that favors oxidation-reduction lability, suggest that the biochemistry of Nrf2 regulation may have evolved to function optimally in the presence of this family of co-factors. Consequently, one or more of the catechol family Nrf2 co-factors actually may be required for proper functioning of Nrf2 defense.

As illustrated in Figs 9–18, several lactobacillus species, in combination with common dietary components, can provide a ready source of 4-ethylcatechol, 4-vinylcatechol, and catechol. Phenolic acid decarboxylase (PAD) (Fig 9) is pivotal to the generation of these Nrf2 co-factors. PAD is expressed by a subset of lactobacillus species found ubiquitously in nature [91, 92, 94, 100, 108]. In particular, many of these lactobacilli are associated with a variety of food products of plant origin that are fermented by traditional, but not modern, methods (reviewed in [93]). These foods and beverages include many fermented vegetables such as olives, cabbage, cucumbers, eggplants, caper berries, and grape extracts (reviewed in [93]), and also traditional ciders [99, 100, 109, 110], wines [108, 111], malt whiskeys [92], and sourdough breads [112]. In addition to fermentative lactobacilli, fermentative yeasts such as the *Brettanomyces/Dekkera* species found in traditional ciders and beers also express PAD [101, 113–115]. Consistent with the presence of PAD-expressing lactobacilli and yeasts in foods fermented by traditional methods, alkyl catechols have been identified in ciders and wines [99, 109, 116], catechol has been identified in fermented cherry juice [95], and 4-vinlycatechol and 4-ethycatechol have been identified in traditional beers fermented with *Brettanomyces* yeasts [117]. To our knowledge, alkyl catechols and catechol have not been isolated from more complex fermented foods such as fermented vegetables; nonetheless, they are likely present in all foods containing caffeic acid or 3,4-dihydroxybenzoic acid and fermented by PAD-expressing lactic acid bacteria and/or yeasts. They are also likely present in in malt whiskeys fermented by PAD-expressing lactobacilli [92].

As illustrated in Fig 15, two additional microbial enzymes, cinnamoyl esterase [100] and phenolic acid reductase (vinylphenol reductase) [100], together with microbial PAD, greatly expand the natural availability of alkyl catechol co-factors. Cinnamoyl esterase liberates caffeic acid from chlorogenic acid, thus providing caffeic acid for conversion by PAD to 4-vinylcatechol. In addition, phenolic acid reductase reduces 4-vinylcatechol to 4-ethylcatechol, which is a more stable compound than 4-vinylcatechol. We demonstrated in our experiments (Figs 16–18) that *L*. *collinoides*, found in traditional ciders [100], performs all of these steps thereby generating 4-ethylcatechol, a potent Nrf2 co-factor, from chlorogenic acid. Thus, chlorogenic acid, which is found in a wide variety of fruits and vegetables and in particularly high concentrations in coffee [102], is an important source of Nrf2 co-factor when incubated with *L*. *collinoides*.

In addition to *L*. *colloinoides*, other lactobacilli also express cinnamoyl esterase including *L*. *helveticus* [118], *L*. *reuteri* [95], and *L*. *johnsonii* [119]. Other lactobacilli also express phenolic acid reductase; examples include *L*. *fermentum* and *L*. *reuteri* [95]. Interestingly, some strains of lactobacilli may express phenolic acid reductase but not PAD (e.g. *L*. *reuteri* [95, 120, 121]), or express PAD but not phenolic acid reductase (e.g., *L*. *plantarum* [93, 95]). Consequently, depending on which lactobacilli or PAD-expressing yeasts are present, multiple microbial strains might be required to achieve the complete bioconversion of chlorogenic acid to 4-ethylcatechol, as accomplished by *L*. *collinoides*. Such “collaborative” bioconversions by multiple strains are entirely plausible and even probable.

It should be noted that caffeic acid, 3,4-dihydroxybenzoic acid, and chlorogenic acid are different from most common dietary phenolic acids with regard to the benzene ring. For example, the benzene ring of ferulic acid, another common phenolic acid, contains two hydroxyls similar to caffeic acid, however one of the hydroxyls is methylated. Coumaric acid, another common phenolic acid, has only one hydroxyl group, whereas cinnamic acid has no hydroxyls. Sinapic acid and syringic acid each have three hydroxyls on the benzene ring, but two are methylated. Consequently, decarboxylation of either ferulic acid, coumaric acid, cinnamic acid, sinapic acid or syringic acid is not expected to yield compounds with oxidation-reduction lability similar to the catechols or quinones [51]. Also *L*. *plantarum* is reportedly unable to demethylate ferulic acid to form caffeic acid [122]. Therefore, caffeic acid and 3,4-dihydroxybenzoic acid, and also chlorogenic acid, appear to be special among common dietary phenolic acids because cleavage of these substrates by PAD can yield potently active catechols.

For plant eating mammals, including early hominids, it seems likely that the earliest source of alkyl catechols and catechol for supporting the Nrf2 pathway was provided by microbial metabolism of dietary precursors directly within the digestive tract. Mammals do not express PAD; therefore generation of 4-vinlycatechol from caffeic acid and catechol from 3,4-dihydroxybenzoic acid (Figs 9–14), is entirely dependent on bacteria expressing the PAD enzyme. Lactobacilli that express PAD, such as *L*. *plantarum*, are commonly associated with plants in the wild; consequently, a diet consisting of raw vegetables and fruits provides both precursor molecules and PAD-expressing bacteria for generation of catechols directly within the digestive tract. Interestingly, 4-methylcatechol and catechol have been detected in the urine of rats fed crude vegetable diets [123]. Also, when caffeic acid was fed to conventional laboratory rats, 4-ethylcatechol was detected in urine; however, in parallel experiments with germ-free rats fed caffeic acid, 4-ethylcatechol was not found [124]. In studies on caffeic acid metabolism *in vitro* by bacteria of the human gastrointestinal tract, mixed cultures of fecal bacteria were found to convert caffeic acid to 4-ethylcatechol [125].

Foods fermented with PAD-expressing lactobacilli [93] likely provide a rich source of bacteria that can generate catechols directly within the digestive tract. The necessity of PAD for generation of Nrf2 co-factors has important implications for the use of modern “probiotics”. Many strains of “probiotic” bacteria do not express PAD, including many strains of lactobacilli. For example, the genome of *Lactobacillus acidophilus*, a popular commercially available probiotic, has been sequenced [126]; and it lacks the phenolic acid decarboxylase gene. In contrast, *L*. *plantarum*, which is also a commercially available probiotic, does indeed express PAD [91] (Figs 9–14). Thus, going forward, it will be important to consider health benefits of various probiotics in the context of PAD and Nrf2 defense.

In addition to microbial enzymes, high temperature also generates alkyl catechols and catechol from common plant products. Catechol, 4-methylcatechol, and 4-ethylcatechol all have been identified in wood smoke condensate [127–131]. Each of these compounds is generated from wood lignins, a major structural component of hard and soft woods, by thermal degradation (pyrolysis); and all likely contribute to the flavor of traditionally smoked foods [127–131]. Anthropologists have estimated that humans may have begun cooking with fire as early as 1.8 million years ago, and it has been postulated that this change in diet corresponded closely with a major increase in human brain size that was a major advance in human evolution [132–135]. It seems probable that cooking with wood fire provided a dietary source of akyl catechols and catechol, an important benefit that has not been considered previously. Moreover, wood smoking of meat and fish, one of the oldest means for food preservation, may have begun shortly after the development of cooking with wood fire. Although the health hazard of wood smoke in poorly ventilated environments has received much attention, it is mostly attributable to airborne fine particles [136] that cause lung disease but do not pose a problem for food. In contrast, alkyl catechols and catechol, imparted to foods by wood smoke, would be expected to provide a health benefit by supporting Nrf2 defense. Interestingly, thermal decomposition of caffeic acid during roasting of coffee also yields 4-vinyl catechol [137] suggesting that thermal roasting of plant materials may have provided an additional early dietary source of catechols. Thus, there is much evidence that both microbial digestion and thermal decomposition of common plant chemicals can provide alkyl catechols and catechol from common plant sources.

In modern times, a variety of changes in food technology have resulted in a precipitous decline in traditional dietary sources of alkyl catechols and catechol. Cooking with wood fire and preservation of meat and fish with wood smoke are relatively rare. Similarly, alkyl catechols and catechol, together with PAD-expressing lactobacilli, have been lost from modern Western foods. Technological advances in canning and refrigeration have caused a marked shift away from foods fermented with traditional methods, particularly those fermented with “wild” lactobacilli and yeasts that express PAD. Undoubtedly, this shift is partly due to the convenience of modern refrigeration and the advantages of food production on an industrial scale. However, the modern perception of undesirable “off-flavors” associated with compounds generated by PAD, such as alkyl catechols and also alkyl phenols, has driven the elimination of PAD-expressing microbes from commercially fermented foods and beverages. For example, 4-ethylcatechol is considered an undesirable off-flavor defect of commercial French ciders that is attributable to the conversion of caffeic acid by two PAD-expressing microbes, *Lactobacillus collinoides* and *Dekkara anomala* (yeast) [99, 101]. Similarly, the presence of PAD-expressing microbes in wine has been linked to serious off-flavors and wine spoilage [114, 138–140]. Prior to modern times, it is unlikely that such off-flavors could have been controlled because PAD-expressing microbes such as *Lactobacillus plantarum* and *Brettanomyces* are ubiquitous in nature. However, modern sterilization techniques and fermentation technology have allowed for stricter control of microbial content and thereby provided the means for eliminating PAD-expressing microbes from the fermentation process.

Thus, several aspects of modern food technology, including the elimination of cooking with wood fire, the shift to food preservation by canning and refrigeration, and the brewing of alcoholic beverages under strictly controlled conditions, all have contributed to the elimination PAD-expressing microbes, together with elimination of alkyl catechols and catechol, from the modern Western diet. Collectively, these large-scale dietary changes have potentially serious negative consequences for Nrf2 defense and health among persons relying exclusively on modern Western foods. In contrast, Asian, Middle Eastern, and African diets still contain many foods prepared and preserved by traditional methods through fermentation with PAD-expressing lactobacilli (reviewed in [141–145]) and thereby continue to provide sources of akyl and catechol Nrf2 co-factors.

## Conclusions

Findings reported here identify akyl catechols as potent natural co-factors for activation of the Nrf2 cellular defense pathway both *in vitro* and *in vivo*. The alkyl catechols and also catechol, another Nrf2 co-factor, are widely available from plant sources but must be generated from these sources either by microbial digestion or heat. Nonetheless, the general availability of these Nrf2 co-factors from numerous natural sources suggests that they are integral to proper functioning of the Nrf2 defense. Until recent times, dietary sources of akyl catechols and catechol were common, but now virtually all are rare in modern commercially available foods due to changes in food preservation and processing. Consequently, findings here illustrate important distinctions between traditional and modern diets, specifically as they relate to Nrf2 defense against disease. Findings here also describe previously undocumented connections between specific compounds found in fruits and vegetables and specific bacteria that convert these compounds to Nrf2 activators. Although there is a general consensus that diets rich in fruits and vegetables are beneficial for health (reviewed in [146, 147]); and, similarly, that probiotic bacteria are beneficial (reviewed in [103, 148, 149]), mechanistic understanding of such benefits is incomplete. In particular, functional connections between specific probiotics and diets rich in fruits and vegetables as they relate to Nrf2 defense have not been defined previously. Thus, findings here provide new molecular framework for a broader understanding of how diets rich in fruit and vegetables, in combination with specific PAD-expressing probiotics, can promote Nrf2 defense, prevent disease, and improve overall health.

## Supporting Information

S1 Fig Cell viability in the presence of catechol, alkyl catechols, and sulforaphane. Human microvascular endothelial cells, astrocytes, and keratinocytes were incubated with the indicated compounds at the indicated doses in complete medium for 24 hours, and cell viability was measured as described in Materials and Methods. Key: Ctrl = control, Cat = catechol, 4MC = 4-methylcatechol, 4VC = 4-vinylcatechol, 4EC = 4-ethylcatechol, SF = sulforaphane. Error bars = +/- S.D; n ≥ 4 for each data point. Viability was not compromised by catechol or the alkyl catechols in either cell type. However, sulforaphane, at a concentration of 30 μM, particularly reduced endothelial cell and astrocyte viability ~ 25%. *Statistical significance*: extremely significant for individual comparisons between control and 30 μM SF (p < 0.001); no statistically significant differences between control and the other compounds or control and 20 μM SF. Because sulforaphane at 20 μM did not compromise viability detectably in either cell type, we used this concentration of sulforaphane in our experiments.
(TIF)

S2 Fig Catechol and the alkyl catechols protect against hydrogen peroxide-induced cell death, consistent with protection against oxidative stress. Human microvascular endothelial cells were pre-incubated in complete medium with 30 μM of catechol (Cat), 4-methylcatechol (4MC), 4-vinylcatechol (4VC), or 4-ethylcatechol (4EC) for 24 hours. (Ctrl) = control without added compound. Next, where indicated, hydrogen peroxide (H_2_O_2_) was added to a final concentration of 100 μM for 16 hours and cells assayed for viability as described in Materials and Methods. Error bars = +/- S.D; n ≥ 4 for each data point. Catechol and the akyl catechols each strongly protected against H_2_O_2_-induced cell death by ~ 50–80%. *Statistical significance*: Individual comparisons between control cells to which H_2_O_2_ was added versus corresponding cells pre-incubated with the individual catechols prior to addition of H_2_O_2_ indicated that protection provided by each of the compounds was extremely significant (p < 0.0001).
(TIF)

S3 Fig Examples of compounds, structurally related to catechols, that do not induce expression of Nrf2 target genes significantly, in comparison with 4-ethylcatechol (see Fig 5 in manuscript for chemical structures). Test compounds were added to human microvascular endothelial cells and RNA isolated 24 hours later for analyses with RT-PCR. Y-axis = (mRNA copies)/(10^6^ 18S rRNA copies). Nrf2 target genes = heme oxygenase-1 (HO-1) and NAD(P)H:quinone oxidoreductase 1 (NQO1). Control mRNA = CD31 (PECAM-1). Error bars = ± standard deviation (S.D.); n ≥ 3 for each data point. Key: (Ctrl) control, (Guaiacol) guaiacol, (1,2 DMB) 1,2-dimethoxybenze, (2M4M) 2-methoxy-4-methylphenol, (4EG) 4-ethylguaiacol, (4EP) 4-ethylphenol, (Orcinol) orcinol, (2,3-DHBA) 2,3-dihydroxybenzoic acid, (3,4 DHPAA) 3,4-dihydroxyphenylacetic acid, (Quercetin) quercetin, (Luteolin) luteolin, (4EC) 4-ethylcatechol = positive control. All compounds were added to a final concentration of 30 μM with the exception of quercetin and luteolin that were added to a final concentration of 20 μM (the maximum tolerated dose). *Statistical significance*: For HO-1 and NQO1 data panels, individual comparisons between Ctrl and all of the other samples (apart from 4EC positive control) indicated that there are no statistically significant differences. In contrast, individual comparisons between 4EC and control and 4EC and each of the other compounds indicated differences that are all extremely statistically significant (p < 0.0001). For CD31: no statistically significant differences.
(TIF)

S4 Fig Examples of lactobacilli that do not biotransform caffeic acid significantly to induce expression of Nrf2 target genes, as demonstrated with RT-PCR. Y-axis = (mRNA copies)/(10^6^ 18S rRNA copies). Human microvascular endothelial cells, 24 hours after addition of test samples: Ctrl = control, CFA = caffeic acid, (L reut + CFA) = supernatant from *L*. *reuteri* (strain MM4-1A; ATCC PTA-6475) incubated with CFA in PBS-glucose and filter-sterilized, (L rum + CFA) = supernatant from *L*. *ruminus* (ATCC 27780) incubated with CFA in PBS-glucose and filter-sterilized, (L para + CFA) = supernatant from *L*. *paracaseii* (ATCC 25302) incubated with CFA in PBS-glucose and filter-sterilized. CFA and lactobacillus incubations with CFA were added to a final concentration corresponding to 30 μM CFA starting material (see Methods). 4EC = 4-ethylcatechol positive control (30 μM). Nrf2 target genes = HO-1, NQO1, G6PD. Control mRNA = CD31. Error bars = ± S.D.; n ≥ 3 for each data point. *Statistical significance*: For HO-1, NQO1, and G6PD data panels, individual comparisons between Ctrl and all of the other samples (apart from 4EC positive control) indicated that there are no statistically significant differences. In contrast, individual comparisons between 4EC and Ctrl and between 4EC and each of the other samples indicated differences that are extremely statistically significant (p< 0.0002). For CD31: no statistically significant differences.
(TIF)

S1 Table Primer Sequences used for RT-PCR. (PDF)

## Abbreviations

3,4-DHBA: 3,4-dihydroxybenzoic acid
CFA: caffeic acid
G6PD: glucose-6-phosphate dehydrogenase
HO-1: heme oxygenase-1
HPLC: high pressure liquid chromatography
i.p.: intraperitoneal
NQO1: NAD(P)H:quinone oxidoreductase1
PAD: phenolic acid decarboxylase
ROS: reactive oxygen species
RT-PCR: real-time polymerase chain reaction
S.D.: standard deviation
siRNA: small interfering RNA
vs.: versus
