# Supplementary figures and images for: Activation of the Nrf2 Cell Defense Pathway by Ancient Foods: Disease Prevention by Important Molecules and Microbes Lost from the Modern Western Diet
Source: PLoS One. 2016 Feb 17;11(2):e0148042. doi: 10.1371/journal.pone.0148042 (PMC4757558; doi:10.1371/journal.pone.0148042)

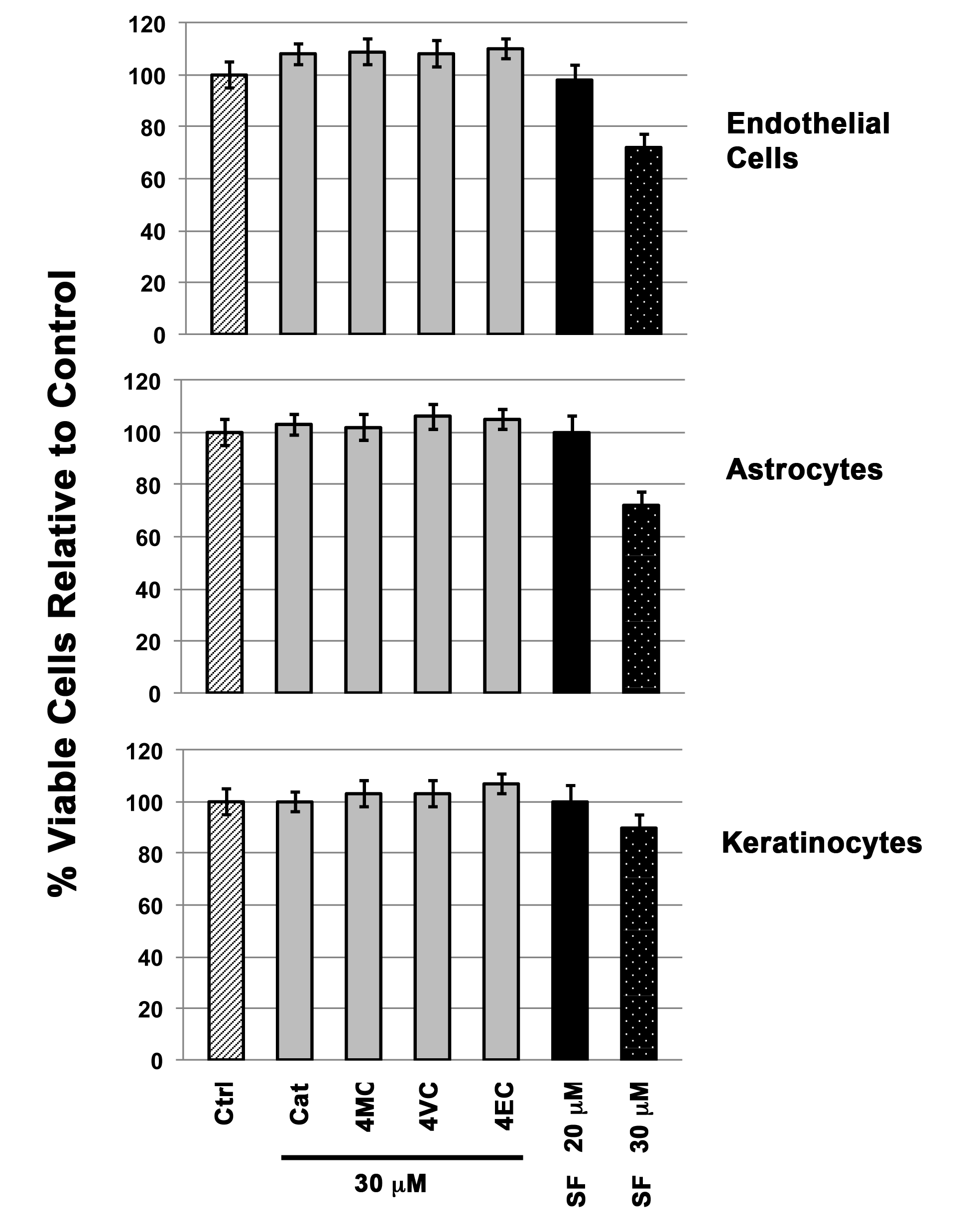

Supplement: S1 Fig — Human microvascular endothelial cells, astrocytes, and keratinocytes were incubated with the indicated compounds at the indicated doses in complete medium for 24 hours, and cell viability was measured as described in Materials and Methods. Key: Ctrl = control, Cat = catechol, 4MC = 4-methylcatechol, 4VC = 4-vinylcatechol, 4EC = 4-ethylcatechol, SF = sulforaphane. Error bars = +/- S.D; n ≥ 4 for each data point. Viability was not compromised by catechol or the alkyl catechols in either cell type. However, sulforaphane, at a concentration of 30 μM, particularly reduced endothelial cell and astrocyte viability ~ 25%. Statistical significance: extremely significant for individual comparisons between control and 30 μM SF (p < 0.001); no statistically significant differences between control and the other compounds or control and 20 μM SF. Because sulforaphane at 20 μM did not compromise viability detectably in either cell type, we used this concentration of sulforaphane in our experiments. (TIF) [file pone.0148042.s001.tif]

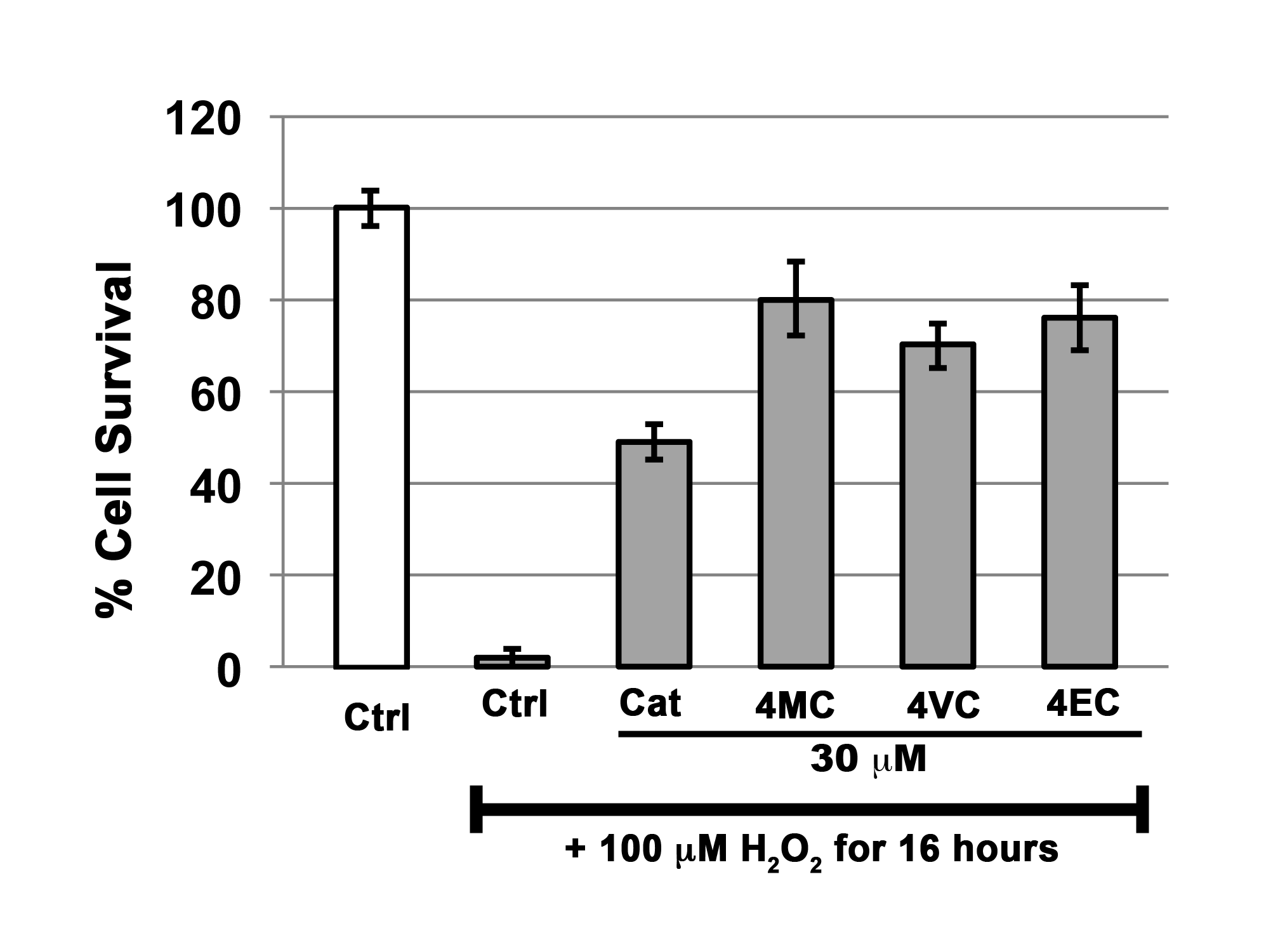

Supplement: S2 Fig — Human microvascular endothelial cells were pre-incubated in complete medium with 30 μM of catechol (Cat), 4-methylcatechol (4MC), 4-vinylcatechol (4VC), or 4-ethylcatechol (4EC) for 24 hours. (Ctrl) = control without added compound. Next, where indicated, hydrogen peroxide (H2O2) was added to a final concentration of 100 μM for 16 hours and cells assayed for viability as described in Materials and Methods. Error bars = +/- S.D; n ≥ 4 for each data point. Catechol and the akyl catechols each strongly protected against H2O2-induced cell death by ~ 50–80%. Statistical significance: Individual comparisons between control cells to which H2O2 was added versus corresponding cells pre-incubated with the individual catechols prior to addition of H2O2 indicated that protection provided by each of the compounds was extremely significant (p < 0.0001). (TIF) [file pone.0148042.s002.tif]

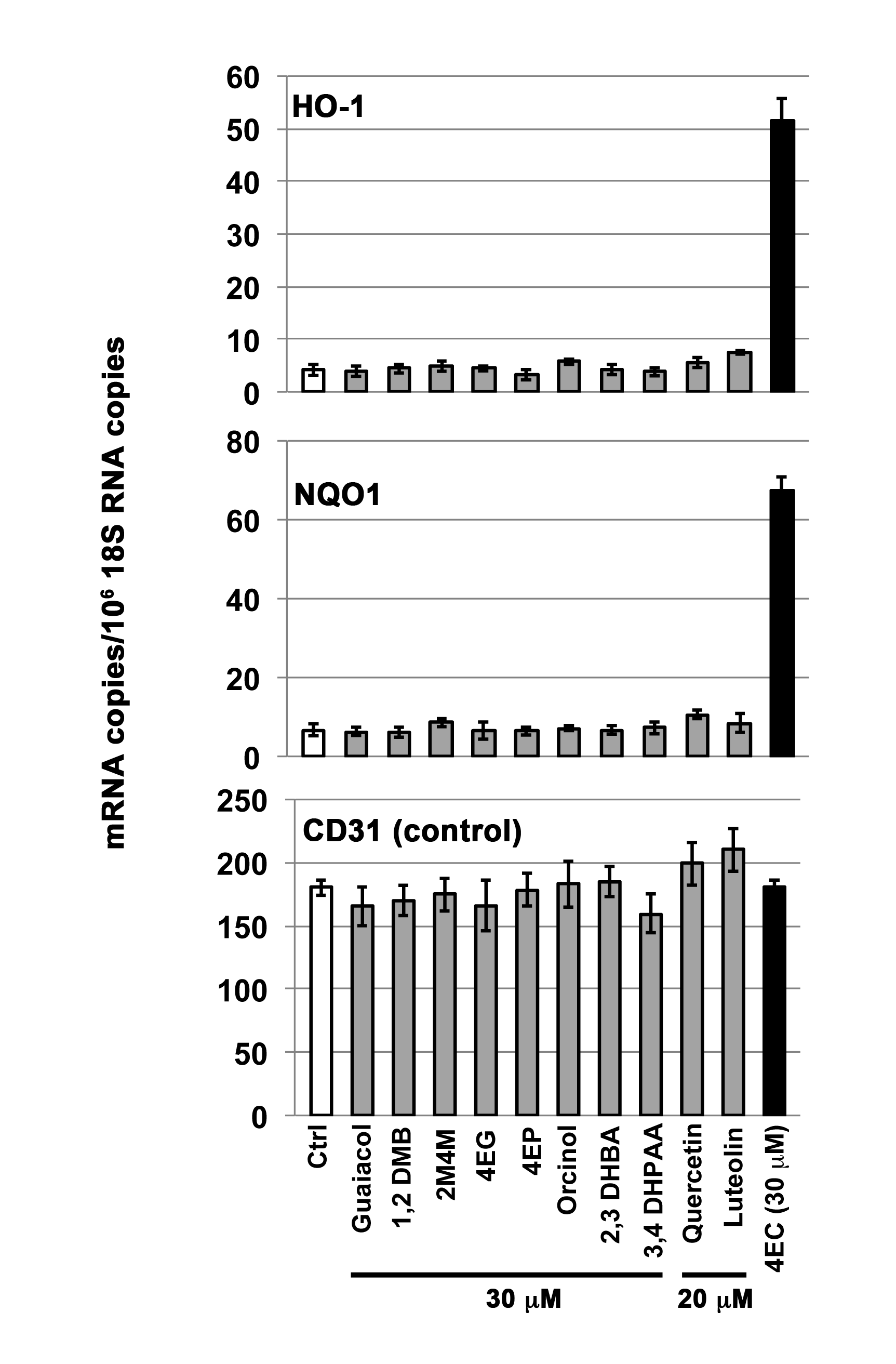

Supplement: S3 Fig — Test compounds were added to human microvascular endothelial cells and RNA isolated 24 hours later for analyses with RT-PCR. Y-axis = (mRNA copies)/(106 18S rRNA copies). Nrf2 target genes = heme oxygenase-1 (HO-1) and NAD(P)H:quinone oxidoreductase 1 (NQO1). Control mRNA = CD31 (PECAM-1). Error bars = ± standard deviation (S.D.); n ≥ 3 for each data point. Key: (Ctrl) control, (Guaiacol) guaiacol, (1,2 DMB) 1,2-dimethoxybenze, (2M4M) 2-methoxy-4-methylphenol, (4EG) 4-ethylguaiacol, (4EP) 4-ethylphenol, (Orcinol) orcinol, (2,3-DHBA) 2,3-dihydroxybenzoic acid, (3,4 DHPAA) 3,4-dihydroxyphenylacetic acid, (Quercetin) quercetin, (Luteolin) luteolin, (4EC) 4-ethylcatechol = positive control. All compounds were added to a final concentration of 30 μM with the exception of quercetin and luteolin that were added to a final concentration of 20 μM (the maximum tolerated dose). Statistical significance: For HO-1 and NQO1 data panels, individual comparisons between Ctrl and all of the other samples (apart from 4EC positive control) indicated that there are no statistically significant differences. In contrast, individual comparisons between 4EC and control and 4EC and each of the other compounds indicated differences that are all extremely statistically significant (p < 0.0001). For CD31: no statistically significant differences. (TIF) [file pone.0148042.s003.tif]

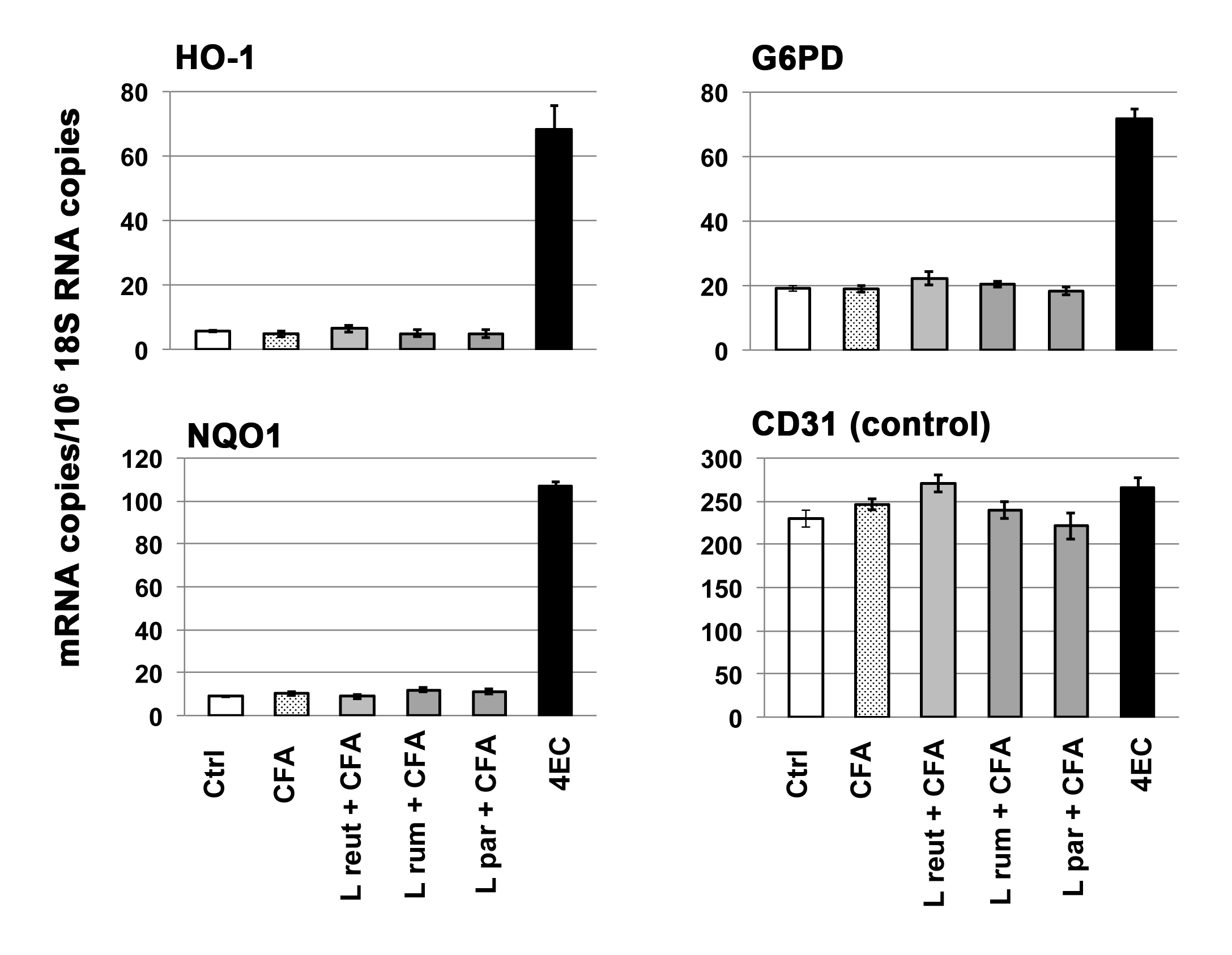

Supplement: S4 Fig — Y-axis = (mRNA copies)/(106 18S rRNA copies). Human microvascular endothelial cells, 24 hours after addition of test samples: Ctrl = control, CFA = caffeic acid, (L reut + CFA) = supernatant from L. reuteri (strain MM4-1A; ATCC PTA-6475) incubated with CFA in PBS-glucose and filter-sterilized, (L rum + CFA) = supernatant from L. ruminus (ATCC 27780) incubated with CFA in PBS-glucose and filter-sterilized, (L para + CFA) = supernatant from L. paracaseii (ATCC 25302) incubated with CFA in PBS-glucose and filter-sterilized. CFA and lactobacillus incubations with CFA were added to a final concentration corresponding to 30 μM CFA starting material (see Methods). 4EC = 4-ethylcatechol positive control (30 μM). Nrf2 target genes = HO-1, NQO1, G6PD. Control mRNA = CD31. Error bars = ± S.D.; n ≥ 3 for each data point. Statistical significance: For HO-1, NQO1, and G6PD data panels, individual comparisons between Ctrl and all of the other samples (apart from 4EC positive control) indicated that there are no statistically significant differences. In contrast, individual comparisons between 4EC and Ctrl and between 4EC and each of the other samples indicated differences that are extremely statistically significant (p< 0.0002). For CD31: no statistically significant differences. (TIF) [file pone.0148042.s004.tif]
