# Supplementary material for: Activation of the Nrf2 Cell Defense Pathway by Ancient Foods: Disease Prevention by Important Molecules and Microbes Lost from the Modern Western Diet
Source: PLoS One. 2016 Feb 17;11(2):e0148042. doi: 10.1371/journal.pone.0148042 (PMC4757558; doi:10.1371/journal.pone.0148042)
Supplement: S1 Table — (PDF) [file pone.0148042.s005.pdf]

Primer sequences used for RT-PCR; F = forward, R= Reverse; Hu = human, Mu = mouse

**Hu CDH5 (CD144; VE-Cadherin).F**	GAACCCAAGATGTGGCCTTTAG
**Hu CDH5 (CD144; VE-Cadherin).R**	GATGTGACAACAGCGAGGTGTAA
**Hu CD31 (PECAM1).F**	CACCTGGCCCAGGAGTTTC
**Hu CD31 (PECAM1).R**	AGTACACAGCCTTGTTGCCATGT
**Hu HMOX1 (HO-1).F**	GCTGAGTTCATGAGGAACTTTCAG
**Hu HMOX1 (HO-1).R**	TGGTACAGGGAGGCCATCAC
**Hu SLC2A1 (GLUT1).F**	TGCTCATGGGCTTCTCGAA
**Hu SLC2A1 (GLUT1).R**	TGGTCAGGCCGCAGTACAC
**Hu NQO1.F**	GCCGCAGACCTTGTGATATTC
**Hu NQO1.R**	TCCTATGAACACTCGCTCAAACC
**Hu CD324 (CDH1, E-cadherin).F**	CTGGGCAGAGTGAATTTTGAAGA
**Hu CD324 (CDH1, E-cadherin).R**	AATCGGGTGTCGAGGGAAA
**Hu G6PD.F**	GCCTTCTGCCCGAAAACAC
**Hu G6PD.R**	TGCGGATGTCAGCCACTGT
**Mu NQO1.F**	CGCCTGAGCCCAGATATTGT
**Mu NQO1.R**	CCACTGCAATGGGAACTGAA
**Mu Hmox1(HO-1).F**	GTGATGCTGACAGAGGAACACAA
**Mu Hmox1(HO-1).R**	GCTAGCAGGCCTCTGACGAA
**Hu NFE2L2(Nrf2).F**	GCATGATGCCCAATGTGAGA
**Hu NFE2L2(Nrf2).R**	TCCAAGCGGCTTGAATGTTT
**Hu CD29 (integrin beta 1).F**	CAACGAGGTCATGGTTCATGTT
**Hu CD29 (integrin beta 1).R**	CCAGCTACAATTGGAATGATGTCT
**Mu Nfe2l2(Nrf2).F**	GAGTCGCTTGCCCTGGATATC
**Mu Nfe2l2(Nrf2).R**	TCATGGCTGCCTCCAGAGA
**Mu ACTB-5UTR (beta actin).F**	GCGAGCACAGCTTCTTTGC
**Mu ACTB-5UTR(beta actin).R**	TCGTCATCCATGGCGAACT
**Hu + Mu 18S rRNA.F**	TGTCTCAAAGATTAAGCCATGCA
**Hu + Mu 18S rRNA.R**	GCGACCAAAGGAACCATAACTG
